# The heritable basis of gene–environment interactions in cardiometabolic traits

**DOI:** 10.1007/s00125-016-4184-0

**Published:** 2016-12-21

**Authors:** Alaitz Poveda, Yan Chen, Anders Brändström, Elisabeth Engberg, Göran Hallmans, Ingegerd Johansson, Frida Renström, Azra Kurbasic, Paul W. Franks

**Affiliations:** 1 Genetic and Molecular Epidemiology Unit, Department of Clinical Sciences, Clinical Research Centre, Lund University, Jan Waldenströms gata 35, Building 91, Skåne University Hospital, SE-20502 Malmö, Sweden; 2 Department of Genetics, Physical Anthropology and Animal Physiology, Faculty of Science and Technology, University of the Basque Country (UPV/EHU), Bilbao, Spain; 3 Centre for Demographic and Ageing Research, Umeå University, Umeå, Sweden; 4 Department of Biobank Research, Umeå University, Umeå, Sweden; 5 Department of Public Health and Clinical Medicine, Section for Medicine, Umeå University, Umeå, Sweden; 6 Department of Nutrition, Harvard T. H. Chan School of Public Health, Boston, MA USA

**Keywords:** Cardiometabolic traits, Environment, Extended pedigrees, Gene, Heritability, Interaction, VIKING study

## Abstract

**Aims/hypothesis:**

Little is known about the heritable basis of gene–environment interactions in humans. We therefore screened multiple cardiometabolic traits to assess the probability that they are influenced by genotype–environment interactions.

**Methods:**

Fourteen established environmental risk exposures and 11 cardiometabolic traits were analysed in the VIKING study, a cohort of 16,430 Swedish adults from 1682 extended pedigrees with available detailed genealogical, phenotypic and demographic information, using a maximum likelihood variance decomposition method in Sequential Oligogenic Linkage Analysis Routines software.

**Results:**

All cardiometabolic traits had statistically significant heritability estimates, with narrow-sense heritabilities (*h* ^2^) ranging from 24% to 47%. Genotype–environment interactions were detected for age and sex (for the majority of traits), physical activity (for triacylglycerols, 2 h glucose and diastolic BP), smoking (for weight), alcohol intake (for weight, BMI and 2 h glucose) and diet pattern (for weight, BMI, glycaemic traits and systolic BP). Genotype–age interactions for weight and systolic BP, genotype–sex interactions for BMI and triacylglycerols and genotype–alcohol intake interactions for weight remained significant after multiple test correction.

**Conclusions/interpretation:**

Age, sex and alcohol intake are likely to be major modifiers of genetic effects for a range of cardiometabolic traits. This information may prove valuable for studies that seek to identify specific loci that modify the effects of lifestyle in cardiometabolic disease.

**Electronic supplementary material:**

The online version of this article (doi:10.1007/s00125-016-4184-0) contains peer-reviewed but unedited supplementary material, which is available to authorised users.

## Introduction

Cardiometabolic diseases are the predominant cause of mortality, morbidity and healthcare spending globally [1, 2], and are believed to result in part from the combined additive and synergistic effects of genetic and environmental risk factors. Environmental exposures such as diet and physical activity have enormous potential for prevention and treatment of these diseases, but no single therapy works well in all individuals. Determining whether susceptibility to adverse environmental exposures is genetically determined (i.e. gene–environment interactions [3]) and elucidating the specific nature of these interactions may facilitate the stratification of patient populations into subgroups that can be treated with optimal therapies.

In contemporary population genetics research, the heritability of a given trait is usually assessed by quantitative genetics approaches to make inferences about the extent to which polygenic variation influences the trait. Assessing heritability is usually done prior to embarking on studies that seek to discover specific loci influencing the trait. While it is equally logical to use quantitative genetics to determine whether traits are influenced by genotype–environment interactions as a prelude to studies focused on specific environmental exposures and genetic loci, this is rarely done in practice [4–8]. The dearth of such studies may be because large, well-characterised cohorts including genealogies, which are necessary for genotype–environment quantitative genetic studies, are rare.

Here we sought to screen for genotype–environment interactions across a number of environmental exposures and cardiometabolic traits using quantitative genetic analyses in extended pedigrees. Accordingly, we characterised the genealogical structure of a large northern Swedish population, within which detailed measures of environmental exposures, cardiometabolic traits and other personal characteristics exist [9].

## Methods

### Study participants

The Västerbotten Imputation Databank of Near-Complete Genomes (VIKING) study is nested in a population-based cohort from the county of Västerbotten in northern Sweden. The study capitalises on the extensively mapped genealogies in this low admixture population, in combination with an ongoing health survey in the population that makes available extensive phenotypic data in the cohort [9]. The genealogical information stems from the POPLINK database at the Demographic Database/Centre for Demographic and Ageing Research (CEDAR) at Umeå University, Umeå, Sweden. Data are based on detailed Swedish population registers, covering the period 1700–1950, linked to population data from Statistics Sweden from 1950 to the present day. Lifestyle and clinical data were collected within the framework of the Västerbottens Health Survey (also called the Västerbottens Intervention Project) initiated in 1985 [10]. In the Västerbottens Health Survey, residents within the county are invited to attend an extensive health examination in the years of their 40th, 50th and 60th birthdays. For the current analysis, health examinations were performed between 1985 and 2013. All participants provided written informed consent as part of the Västerbottens Health Survey, and the study was approved by the regional ethics review board in Umeå, Sweden.

The current study includes 1682 extended pedigrees comprising 193,060 people of whom 16,430 have detailed phenotype data. The most extended genealogy descends from 4255 founders and contains 160,533 people of whom 10,498 are phenotyped. The phenotyped sample includes 8908 first-degree relative pairs, 5794 second-degree relative pairs and 29,706 third-degree relative pairs, in addition to other more distant relatives (electronic supplementary material [ESM] Table 1).

### Cardiometabolic traits

The assessment of clinical measures in the Västerbottens Health Survey has been described in detail elsewhere [10, 11]. Briefly, weight (to the nearest 0.1 kg) and height (to the nearest 1 cm) were measured with a calibrated balance-beam scale and a wall-mounted stadiometer, respectively, and with participants wearing indoor clothing and without shoes. BMI was calculated as weight (kg)/height (m)^2^. In a subgroup, waist circumference was measured using a non-stretchable nylon tape at the midpoint between the 12th rib and the iliac crest. Systolic and diastolic BPs (SBP and DBP) were measured once using a mercury sphygmomanometer following a 5 min rest. Capillary blood was drawn after an overnight fast and again 2 h after administration of a standard 75 g oral glucose load [12]. Before the first blood draw, 83% of participants had fasted for a minimum of 8 h. Capillary plasma glucose concentrations, total cholesterol and triacylglycerols were measured with a Reflotron bench-top analyser (Roche Diagnostics Scandinavia, Umeå, Sweden). HDL-cholesterol (HDL-C) was measured in a subgroup of participants. LDL-cholesterol (LDL-C) was calculated by applying the Friedewald formula: LDL-C=total cholesterol−HDL-C−(triacylglycerol/2.2) [13]. The analysis methods for total cholesterol, triacylglycerol, SBP and DBP changed in 2009: from Reflotron to a clinical chemical analysis at the laboratory for total cholesterol and triacylglycerol, and from BP measurements taken once in the supine position to being taken twice in a sitting position (the average of these two values being used in analyses). Lipid and BP values taken after 2009 were therefore corrected to make them comparable to values taken before 2009. Lipid and BP traits were also corrected for the use of lipid-lowering and antihypertensive medication using published constants (total cholesterol +1.347 mmol/l, triacylglycerol +0.208 mmol/l, HDL-C −0.060 mmol/l, LDL-C +1.290 mmol/l, SBP +15 mmHg, DBP +10 mmHg) [14, 15].

### Lifestyle assessment

Participants completed a self-administered questionnaire that queried physical activity levels and diet and asked additional questions about tobacco use and alcohol consumption. Diet was assessed using a validated semi-quantitative food-frequency questionnaire designed to capture habitual dietary intake over the last year [16, 17]. The initial food-frequency questionnaire (used from 1985) covered 84 independent or aggregated food items but was reduced in 1996 to 66 food items by combining several questions related to similar foods and deleting some. Participants with ≥10% of the food-frequency questionnaire missing or a seemingly implausible total energy intake (<2093 or >18,841 kJ/day; <500 or >4500 kcal/day) were excluded from the analyses.

In order to obtain a summary factor representing the overall dietary pattern, a principal component analysis including all macronutrients (i.e. carbohydrate, protein, total fat, saturated fat, monounsaturated fatty acids [MUFA], polyunsaturated fatty acids [PUFA], essential fatty acids [*n*-3 and *n*-6 fatty acids], and fibre intakes expressed as per cent of total energy intake [E%]) was conducted, as previously described [18]. A single factor that contrasted carbohydrate and fibre intake against fat intake and accounted for 53.8% of the variance of all macronutrients was retained (ESM Table 2).

A validated modified version of the International Physical Activity Questionnaire [19] was used to gather information on leisure time physical activity for the past 3 months categorised as: (1) never; (2) occasionally; (3) 1–2 times/week; (4) 2–3 times/week; or (5) more than 3 times/week. For the current analyses, categories were combined into physically inactive (never and occasionally) and physically active (≥1–2 times/week).

### Statistical analyses

All cardiometabolic traits were first adjusted for age, age^2^, sex and their interactions (age–sex and age^2^–sex) by conducting a multiple regression analysis using R software (version 3.1.1) [20] and retaining the residuals. Models with glycaemic and lipid traits as the dependent variables were additionally adjusted for fasting status. Models were also adjusted for the environmental exposure that was later tested in the genotype–environment interaction analyses; when the environmental exposure was alcohol intake or a dietary variable the model was also adjusted for the food-frequency questionnaire version. Retained residuals were then normalised by inverse normal transformation and used in the subsequent quantitative genetic analyses as recommended elsewhere [21, 22].

#### Kinship matrix

Kinship coefficients of the 16,430 participants with phenotype data were obtained based on the genealogical information gathered for the whole sample (193,060 individuals) using the CFC program [23], as Sequential Oligogenic Linkage Analysis Routines (SOLAR [24]) software is not designed to analyse such a large sample size.

#### Heritability estimation

Quantitative genetic analyses were conducted using the maximum likelihood-based variance components decomposition method implemented in SOLAR.

In the standard model, the observed covariance of a complex trait (Ω, cardiometabolic trait), assuming that dominance and epistasis are negligible, is defined as:

1 $$ \Omega =2{\Phi \upsigma}_G^2+I{\upsigma}_E^2 $$

Here, Ω is an *N*-by-*N* matrix of the observed covariance of the cardiometabolic trait for each pair of the *N* individuals in the dataset, 2Φ gives the expected coefficient of relationship (Φ, kinship coefficient), σ_*G*_^2^ is the additive genetic variance (i.e. genetic variation attributed to additive effects of the multiple genes affecting the cardiometabolic trait), *I* is the identity matrix of the unique unshared environmental component and σ_*E*_^2^ is the environmental variance. This model is used to estimate narrow-sense heritability (*h* ^2^), i.e. the proportion of the cardiometabolic trait variance attributable to additive genetic effects:

2 $$ {h}^2=\frac{\upsigma_G^2}{\upsigma_G^2 + {\upsigma}_E^2} = \frac{\upsigma_G^2}{\upsigma_P^2} $$

where σ_P_^2^ is the total cardiometabolic trait variance.

#### Genotype–environment interactions

Genotype–environment interactions describe a relationship between genetic variation and changes in the cardiometabolic trait that is conditional on an environmental exposure. The presence of genotype–environment interactions can be tested with an extension of the standard model [equation (1)] [24, 25], which can be adapted for both discrete and continuous environmental exposures [5].

- *For a discrete (dichotomous) environmental exposure:* Adaptation can be made by modelling environment-specific additive genetic and environmental standard deviations and a genetic correlation across the two exposure groups (i.e.. the proportion of variance in a trait explained by the same genetic factors in the two different exposure groups):
  3 $$ \Omega =2\upphi {\uprho}_G{\upsigma}_{G1}{\upsigma}_{G2}+I{\upsigma}_{E1}{\upsigma}_{E2} $$
  Additive genetic variance σ_*G*_^2^ in equation (1) is decomposed as a product of additive genetic standard deviations for the two different environmental exposure groups (${\upsigma}_{G_1}$ and ${\upsigma}_{G_2}$) and a genetic correlation across the two groups denoted by ρ_*G*_ [7, 25], i.e. ${\upsigma}_G^2={\uprho}_G{\upsigma}_{G_1}{\upsigma}_{G_2}$. In the same way, environmental variance is decomposed into the environmental standard deviations for the two different environmental groups (${\upsigma}_{E_1}$ and ${\upsigma}_{E_2}$), i.e. σ_*E*_^2^ = σ_*E*1_σ_*E*2_. Because the statistical genetic model assumes that the genetic and environmental effect estimates are uncorrelated, the function does not include an environmental correlation term. In the presence of genotype–environment interactions, narrow-sense heritability in k-th (k = 1,2) discrete environmental exposure group can then be estimated as: ${h}_{Ek}^2=\frac{\upsigma_{Gk}^2}{\upsigma_{Gk}^2+{\upsigma}_{Ek}^2}$ [7].
- *For a continuous environmental exposure:* Both additive genetic variance σ_*G*_^2^ and genetic correlation ρ_*G*_ can be modelled as exponential functions of the levels of the continuous environmental exposure [5, 26]. Genetic variance is modelled as:
  4 $$ {\upsigma}_G^2= \exp \left({\upalpha}_G+{\upgamma}_G\left({e}_i-\overline{e}\right)\right) $$
  where α_*G*_ and γ_*G*_ are parameters to be estimated, and *e*_*i*_ is the value of the environmental exposure *e* of the i-th individual standardised against the sample mean (*ē*). Genetic correlation is modelled as an exponential decay function of the absolute difference of the pair-wise environmental exposure differences for the *i*-th and *j*-th individuals as:
  5 $$ {\uprho}_G= \exp \left(-\uplambda \left|{e}_i-{e}_j\right|\right) $$
  where λ is the parameter to be estimated.

The null hypothesis of genotype–environment interaction is that the expression of the genotype is independent of the environment. It can be shown that in the absence of a genotype–environment interaction (null hypothesis): (1) the genetic variance (σ_*G*_^2^) will be homogenous across the levels of environmental exposure; and (2) the same quantitative trait measured in participants living in different levels of environmental exposure (e.g. active vs inactive or different ages) will have a genetic correlation (ρ_*G*_) of 1.0 [5, 25, 27]. Hence, the presence of genotype–environment interactions is determined by testing two null hypotheses, which for the sake of simplicity will be referred to as class 1 and class 2 interactions from here on.

- *Class 1 interaction:* The extended model is restricted by assuming homogenous genetic variance (σ_*G*_^2^) across the levels of the environmental exposure. For a discrete environmental exposure [equation (3)], this means that the genetic standard deviations in the two exposure groups are equal, i.e. ${\upsigma}_{G_1}={\upsigma}_{G_2}$. For a continuous environmental exposure [equation (4)], genetic variance (σ_*G*_^2^) is homogenous across the different environmental levels when it is independent of the level of the environmental exposure, i.e. γ_*G*_ = 0.
  Rejection of the model constraining the genetic variance of the groups to be equal (i.e. presence of a significant class 1 interaction) would imply that the magnitude of the genetic effect on the cardiometabolic trait is significantly different depending on the level of the environmental exposure.
- *Class 2 interaction:* The extended model is restricted by constraining the genetic correlation to 1. For a discrete environmental exposure [equation (3)], this means that the same cardiometabolic trait measured in individuals living in the different levels of the environmental exposure will have a genetic correlation of 1.0, i.e. ρ_*G*_ = 1. For a continuous environmental exposure [equation (5)], genetic correlation (ρ_*G*_) is equal to 1.0 if: (1) individuals *i* and *j* have the same level of the environmental exposure; or (2) λ=0. Thus, the null hypothesis of a class 2 interaction (i.e. genetic correlation is equal to 1) is equivalent to λ=0.
  Rejection of the model constraining the genetic correlation between the environmental exposure groups to equal 1 (i.e. presence of a significant class 2 interaction) implies that a different gene or different set of genes are contributing to the variance of the cardiometabolic trait depending on the level of the environmental exposure.

To test the null hypothesis, each restricted model is compared with the extended model using the likelihood ratio test (LRT). The LRT statistic to test the null hypothesis of variance homogeneity (${\upsigma}_{G_1}={\upsigma}_{G_2}$ or γ_*G*_ = 0) is distributed as a χ^2^ random variable with one degree of freedom (χ_1_^2^); the LRT to test the null hypothesis of genetic correlation equal to 1 (ρ_*G*_ = 1 or λ = 0) is distributed as a 50:50 mixture of a χ^2^ random variable with a point mass at zero and one degree of freedom (0.5χ_0_^2^ + 0.5χ_1_^2^) [5].

In the figures representing class 1 and class 2 interactions for continuous environmental exposures, additive genetic variances and genetic correlations were calculated based on equations (4) and (5) and the estimates obtained for α_*G*_, γ_*G*_ and λ parameters.

#### Multiple testing correction

The Bonferroni method assumes that the individual tests are independent of each other. However, the tests conducted in this study were not independent, so we estimated the total number of effective cardiometabolic traits and environmental exposures by accounting for the collective correlation of each set of clinical and environmental variables [28, 29]. The method utilises the estimates of variance of the eigenvalues (λs) derived from the correlation matrix of the set of variables and uses the following formula:

6 $$ {M}_{eff}=1+\left(M-1\right)\left(1-\frac{Var\ \left({\uplambda}_{obs}\right)}{M}\right) $$

where *M*_*eff*_ is the number of effective factors and *M* is the total number of variables (either clinical or environmental) included in the correlation matrix.

For the 14 environmental exposures and 11 cardiometabolic traits, 12.407 and 10.507 effective factors were obtained, respectively. Considering that we tested for both class 1 and class 2 interactions, the total number of effective tests are 260.721 (12.407 × 10.507 × 2). The Bonferroni corrected level of statistical significance for a threshold of 0.05 is thus 0.00019 (0.05/260.721).

## Results

The characteristics of the 16,430 study participants are presented in Table 1.

**Table 1:** Phenotypic and lifestyle characteristics of VIKING study participants (*N* = 16,430)

Trait	Men		Women		All	
	*N*	Mean ± SD or %	*N*	Mean ± SD or %	*N*	Mean ± SD or %
Age, years	7983	52.5 ± 7.8	8447	52.7 ± 7.5	16,430	52.6 ± 7.7
Cigarette smoking, % (current/non-smokers)	4814	35.9/64.1	5944	33.5/66.5	10,758	34.6/65.4
Physical activity, % (inactive/active)	6967	71.1/28.9	7485	71.3/28.7	14,452	71.2/28.8
Fasting status, % (<8 h/≥8 h)	7275	18.1/81.9	7433	16.7/83.3	14,708	17.4/82.6
Height, cm	7927	177.8 ± 6.5	8369	164.3 ± 5.8	16,296	170.8 ± 9.1
Weight, kg	7929	82.4 ± 11.9	8367	69.2 ± 12.3	16,296	75.6 ± 13.8
BMI, kg/m^2^	7923	26.1 ± 3.4	8358	25.7 ± 4.4	16,281	25.8 ± 4.0
Waist circumference, cm	1066	97.6 ± 10.2	839	88.2 ± 12.5	1905	93.5 ± 12.2
TC, mmol/l	7810	5.9 ± 1.2	8251	5.9 ± 1.2	16,061	5.9 ± 1.2
HDL-C, mmol/l	1901	1.3 ± 0.6	1833	1.5 ± 0.6	3734	1.4 ± 0.6
LDL-C, mmol/l	1773	4.3 ± 1.2	1721	4.3 ± 1.2	3494	4.3 ± 1.2
TG, mmol/l	6006	1.6 ± 1.0	6968	1.4 ± 0.8	12,974	1.5 ± 0.9
Fasting glucose, mmol/l	7867	5.6 ± 1.2	8335	5.4 ± 1.0	16,202	5.5 ± 1.1
2 h glucose, mmol/l	7243	6.5 ± 1.9	7778	7.0 ± 1.7	15,021	6.8 ± 1.8
SBP, mmHg	7836	133.9 ± 19.7	8261	132.1 ± 21.1	16,097	133.0 ± 20.4
DBP, mmHg	7833	83.8 ± 12.0	8259	81.0 ± 11.8	16,092	82.4 ± 12.0
Carbohydrate intake, %E	6110	48.5 ± 6.3	6596	51.4 ± 6.0	12,706	50.0 ± 6.3
Protein intake, %E	6110	14.1 ± 2.1	6596	14.9 ± 2.1	12,706	14.5 ± 2.2
Fibre intake, %E	6110	2.1 ± 0.6	6596	2.5 ± 0.6	12,706	2.3 ± 0.6
Total fat intake, %E	6110	35.0 ± 6.2	6596	31.5 ± 5.7	12,706	33.2 ± 6.2
Saturated fat intake, %E	6110	14.7 ± 3.4	6596	13.3 ± 3.1	12,706	14.0 ± 3.3
Essential fatty acid intake, %E^a^	6110	4.7 ± 1.7	6596	4.3 ± 1.4	12,706	4.5 ± 1.6
MUFA intake, %E	6110	11.8 ± 2.4	6596	10.8 ± 2.0	12,706	11.3 ± 2.3
PUFA intake, %E	6110	5.2 ± 1.8	6596	4.8 ± 1.4	12,706	5.0 ± 1.6
Alcohol intake, %E	6110	2.2 ± 2.2	6596	1.3 ± 1.6	12,706	1.7 ± 2.0

Data are expressed as mean ± SD for quantitative variables and as per cent for qualitative variables

^a^Intake of *n*-3 and *n*-6 fatty acids

TC, total cholesterol; TG, triacylglycerol

### Heritability estimates

All 11 cardiometabolic traits showed statistically significant narrow-sense heritability estimates (*h* ^2^ range 0.24–0.47; *p* < 0.001) (Table 2). Waist circumference conveyed the highest heritability estimate, followed by the remaining anthropometric, lipid and BP traits. Glycaemic traits conveyed the lowest heritability estimates.

**Table 2:** Heritability estimates (*h* ^2^) of cardiometabolic traits and per cent of cardiometabolic trait variance attributed to covariate effects (*R* ^2^)

Cardiometabolic trait	*N*	*h* ^2^	SE	*p* value	Per cent of cardiometabolic trait variance attributed to covariate effects (*R* ^2^)
Weight, kg	16,296	0.35	0.01	9 × 10^−118^	23.27
BMI, kg/m^2^	16,281	0.38	0.02	2 × 10^−104^	2.90
Waist circumference, cm	1905	0.47	0.13	3 × 10^−4^	15.30
TC, mmol/l	14,366	0.36	0.02	8 × 10^−96^	7.42
HDL-C, mmol/l	3266	0.37	0.07	8 × 10^−9^	3.24
LDL-C, mmol/l	3169	0.39	0.07	1 × 10^−9^	5.78
TG, mmol/l	11,681	0.35	0.03	4 × 10^−52^	3.00
Fasting glucose, mmol/l	14,507	0.24	0.02	8 × 10^−45^	4.32
2 h glucose, mmol/l	13,603	0.25	0.02	2 × 10^−39^	6.41
SBP, mmHg	16,097	0.33	0.02	1 × 10^−62^	14.69
DBP, mmHg	16,092	0.30	0.02	2 × 10^−53^	8.58

TC, total cholesterol; TG, triacylglycerol

### Genotype–environment interactions

**Fig. 1:**
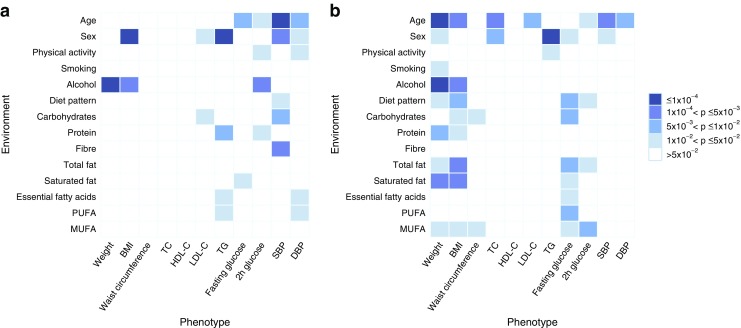
Heat plot showing *p* values for (**a**) class 1 and (**b**) class 2 interactions. Experiment-wise significance threshold is *p* ≤ 1 × 10^−4^ (darkest blue in the heat plot). All environmental exposures are continuous variables except for sex, physical activity and smoking, which are dichotomous variables. TC, total cholesterol; TG, triacylglycerol

To test whether cardiometabolic traits are modulated by genotype–environment interactions the full model was compared with its constrained alternatives (i.e. genetic variance homogeneity and genetic correlation equal to 1). Statistically significant class 1 and class 2 interactions are summarised in Fig. 1.

#### Genotype–age interactions

**Fig. 2:**
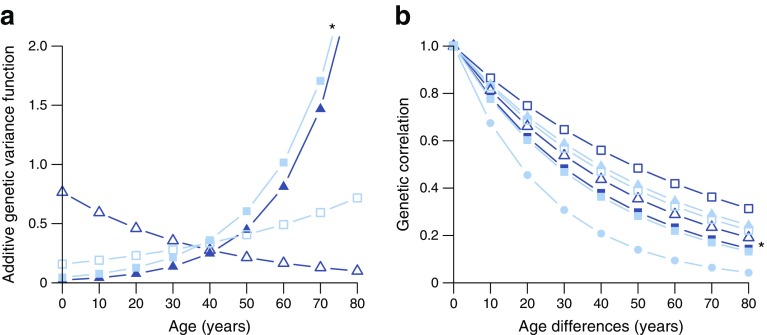
Genotype–age interactions: (**a**) class 1 and (**b**) class 2 interactions. Dark blue full square, weight; dark blue empty square, BMI; dark blue full triangle, fasting glucose; dark blue empty triangle, 2 h glucose; light blue full square, SBP; light blue empty square, DBP; light blue full triangle, total cholesterol; light blue full circle, LDL-C. Only significant traits are represented in the figure. Experiment-wise significant interactions (*p* ≤ 1 × 10^−4^) are marked with an asterisk (SBP for class 1 interactions, and weight for class 2 interactions). *α*_*G*_, *γ*_*G*_ and *λ* parameters were calculated based on individuals 30–60 years of age, as this was the age range in the dataset. A broader age range curve (0–80 years) based on estimates above is displayed in the x-axis to improve the visualisation

All the cardiometabolic traits except waist circumference, HDL-C and triacylglycerol showed significant genotype–age interactions. For fasting glucose, 2 h glucose, SBP and DBP significant class 1 interactions were observed (Fig. 2a). Class 2 interactions were observed for weight, BMI, total cholesterol, LDL-C, 2 h glucose, SBP and DBP (Fig. 2b), suggesting that different sets of genes influence the index traits in older compared with younger participants (ESM Table 3).

#### Genotype–sex interactions

**Fig. 3:**
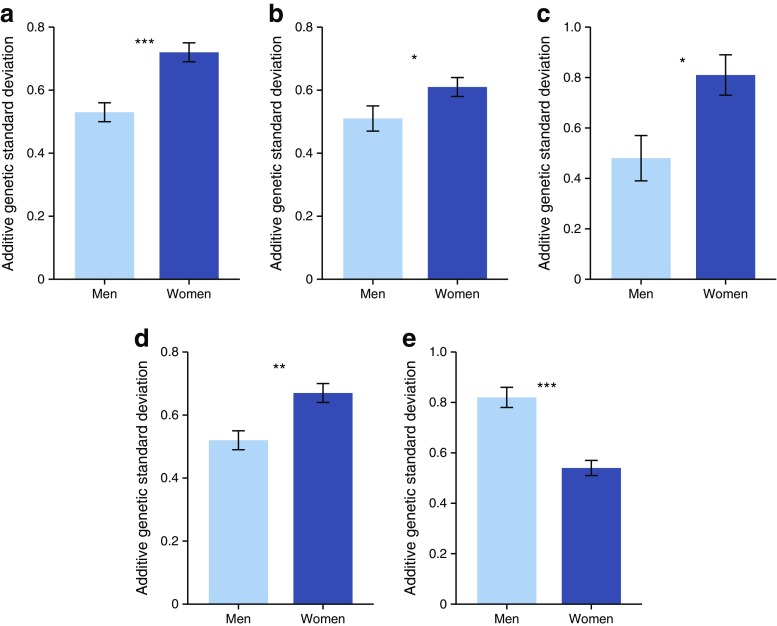
Class 1 genotype–sex interactions for (**a**) BMI, (**b**) DBP, (**c**) LDL-C, (**d**) SBP and (**e**) triacylglycerol. Only significant traits are represented in the figure; *p < 0.05, **p < 0.01, ***p < 0.001. Experiment-wise significant class 1 interactions (*p* ≤ 1 × 10^−4^) are BMI (*p* ≤ 4 × 10^−6^) and triacylglycerol (*p* ≤ 4 × 10^−7^)

Genotype–sex interactions were observed for eight of the 11 cardiometabolic traits. For BMI, LDL-C, triacylglycerol, SBP and DBP class 1 interactions were observed (Fig. 3). The additive genetic effects for BMI, DBP, LDL-C and SBP were greater in women than in men, suggesting that the expression of these cardiometabolic traits is under greater genetic influence in women than in men (*h* ^2^ = 0.44, 0.38, 0.68 and 0.43 in women vs 0.35, 0.26, 0.23 and 0.29 in men for BMI, DBP, LDL-C and SBP, respectively). The additive genetic effects for triacylglycerol were greater in men than in women (*h* ^2^ = 0.49 in men vs 0.44 in women). Class 2 interactions were observed for body weight (ρ_*G*_ = 0.86 ± 0.08; *p* = 0.049), total cholesterol (ρ_*G*_ = 0.79 ± 0.08; *p* = 0.008), triacylglycerol (ρ_*G*_ = 0.55 ± 0.07; *p* = 2 × 10^−10^), fasting glucose (ρ_*G*_ = 0.73 ± 0.13; *p* = 0.03) and SBP (ρ_*G*_ = 0.84 ± 0.08; *p* = 0.03) (ESM Table 4).

#### Genotype–physical activity interactions

Class 1 interactions were observed for DBP and 2 h glucose, with the estimated heritabilities being higher in physically inactive (*h* ^2^ = 0.36 and 0.28, respectively) than in active individuals (*h* ^2^ = 0.20 and 0.16, respectively) (ESM Fig. 1). A class 2 interaction was observed for triacylglycerol (ρ_*G*_ = 0.77 ± 0.11; *p* = 0.03) (ESM Table 5).

#### Genotype–smoking interactions

A class 2 genotype–smoking interaction was observed for body weight (ρ_*G*_ = 0.79 ± 0.11; *p* = 0.04) (ESM Table 6).

#### Genotype–alcohol intake interactions

**Fig. 4:**
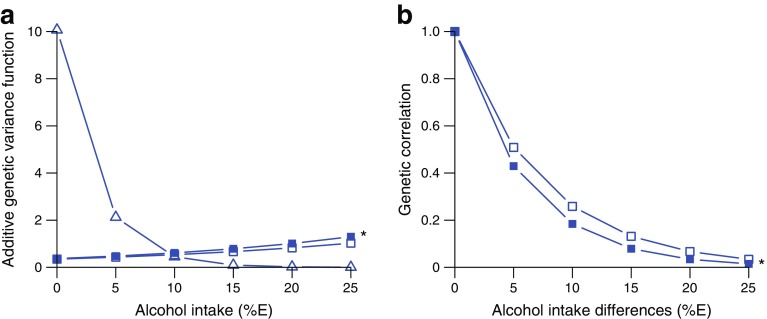
Genotype–alcohol intake interactions: (**a**) class 1 and (**b**) class 2 interactions. Dark blue full square, weight; dark blue empty square, BMI; dark blue empty triangle, 2 h glucose. Only significant traits are represented in the figure. Experiment-wise significant interactions (*p* ≤ 1 × 10^−4^) are marked with an asterisk (weight for class 1 and class 2 interactions)

Body weight, BMI and 2 h glucose concentrations were influenced by genotype–alcohol intake interactions. For 2 h glucose, the interaction was a class 1 interaction (Fig. 4). Both class 1 and class 2 interactions were observed for body weight and BMI, suggesting that the interaction is a joint function of genetic effects that differ in magnitude and of different sets of genes influencing the body composition traits at different levels of alcohol intake (ESM Table 7).

#### Genotype–diet interactions

In order to quantify genotype–diet interactions, we constructed a score representing the global dietary intake (i.e. diet pattern), as described in the Methods section. In a second step, we analysed the interactions with each macronutrient intake variable separately.

#### *Genotype–diet pattern interactions*

Body weight, BMI, glycaemic traits and SBP were influenced by genotype–diet pattern interactions. For SBP, the additive genetic variance decreased as the dietary fat/carbohydrate–fibre ratio increased (class 1 interaction) (ESM Fig. 2a). Class 2 genotype–diet pattern interactions were observed for body weight, BMI, and fasting and 2 h glucose concentrations (ESM Fig. 2b; ESM Table 8).

#### *Genotype–carbohydrate intake interactions*

LDL-C and SBP showed class 1 genotype–carbohydrate intake interactions (ESM Fig. 2c), whereas class 2 genotype–carbohydrate intake interactions were observed for BMI, waist circumference and fasting glucose (ESM Fig. 2d; ESM Table 9).

#### *Genotype–protein intake interactions*

For triacylglycerol and 2 h glucose, class 1 genotype–protein intake interactions were inferred (ESM Fig. 2e). For body weight and BMI, class 2 genotype–protein intake interactions were observed (ESM Fig. 2f; ESM Table 10).

#### *Genotype–fibre intake interactions*

SBP was the only cardiometabolic trait where a genotype–fibre intake interaction (class 1) was evident (ESM Fig. 2g; ESM Table 11).

#### *Genotype–fat intake interactions*

Body weight, BMI, fasting glucose and 2 h glucose showed significant genotype–total fat intake interactions (class 2) (ESM Fig. 3a; ESM Table 12).

Apart from total fat intake, four additional fat intake variables were analysed (saturated fat, essential fatty acids, PUFA and MUFA). Fasting glucose showed a class 1 genotype–saturated fat interaction (ESM Fig. 3b; ESM Table 13). Body weight, BMI and fasting glucose showed a significant class 2 genotype–saturated fat intake interaction (ESM Fig. 3c). Triacylglycerol, fasting glucose and DBP showed significant genotype–essential fatty acids and genotype–PUFA interactions. For triacylglycerol and DBP, the interactions were class 1 interactions, whereas for fasting glucose these interactions were class 2 (ESM Fig. 3d–g; ESM Tables 14 and 15). All anthropometric and glycaemic traits showed significant genotype–MUFA interactions, all of which were class 2 interactions (ESM Fig. 3h; ESM Table 16).

### Multiple testing correction

Seven analyses withstood multiple testing correction: genotype–age interactions for body weight (class 2) and SBP (class 1); genotype–sex interactions for BMI (class 1) and triacylglycerol (class 1 and class 2) and genotype–alcohol intake interactions for body weight (class 1 and class 2).

There was no material change to the interpretation of these results when participants who were not fully fasted were excluded from the interaction analyses for lipid and glycaemic traits (ESM Table 17).

## Discussion

To our knowledge, this is the first compendium of genotype–environment interactions for cardiometabolic traits to be reported. The purpose of doing so is to provide a foundation for subsequent locus-specific analyses of interaction effects and to aid the interpretation of published locus-specific interaction studies. After accounting for multiple testing, we observed robust evidence of genotype–age interactions for body weight and SBP, genotype–sex interactions for BMI and triacylglycerol, and genotype–alcohol intake interaction for body weight.

There are many published reports concerning interactions of environmental exposures with genetic factors in cardiometabolic traits (reviewed in [30–33]). Approaches include quantitative genetics studies, usually undertaken in twin or family-based cohorts [4, 6, 7, 34–38] and candidate gene studies, focused on individual genetic variants, haplotypes, or genetic risk scores constructed from variants with high biological priors for interactions or those conveying genome-wide significant marginal effects [39–47]. Several quantitative genetic studies have shown that physical activity attenuates the influence of genetic effects on cardiometabolic traits [4, 6, 34, 35, 37, 38]. However, only *FTO*–physical activity interactions in obesity [39–42] have been adequately replicated in candidate gene studies. In the present study, we observed evidence of genotype–physical activity interactions for DBP, 2 h glucose (class 1) and triacylglycerol (class 2), but not for obesity-related traits. This may be because analyses of the kind reported here account for the overall modifying effect of genetic variation (polygenic interactions), whereas gene–physical activity interactions in obesity may be oligogenic in nature.

According to our analyses, variation in the intake of macronutrients (whether modelled together or separately) may interact with genetic variation to affect body composition and glycaemic control. Although several candidate gene studies have focused on gene–diet interactions (e.g. [43–47]), there are few quantitative genetics studies on this topic, and these were restricted in scope and conducted in relatively small cohorts [35]. On the other hand, family-based studies have reported class 2 genotype–smoking interactions with serum leptin levels (an important endophenotype of adiposity) [7, 36], and those findings are consistent with the current analyses for body weight.

Although this is a hypothesis-generating study, and as such one might argue against multiple test adjustments owing to the risk of a type II error [48], we adopted a conservative approach to minimise the number of false-positives reported. Nevertheless, as described in the Results section, many of the statistical models yielded nominal evidence of interactions for the environmental exposures and cardiometabolic traits assessed. We present those findings, as the approach used here is orthogonal to standard approaches used to model genotype–environment interactions; thus, the combination of these approaches may help verify the presence or absence of interaction effects. Despite the relatively large sample size used here, it is of course likely that some of the hypothesis tests were underpowered. Statistical power may be diminished by the imprecise nature of the self-reported methods used to assess many of the environmental exposures and the need to dichotomise some of these variables for analysis. Survival bias is a further possible limitation, as people with the most deleterious genetic and/or environmental risk characteristics might have been excluded from the cohort because of early mortality. Systematic error (bias), on the other hand, may lead to false-positive or false-negative conclusions: for example, if an environmental exposure is over-reported at high or low levels of the cardiometabolic trait, or a strong correlate [49], an observed genotype–environment interaction may be false-positive. However, this limitation clearly does not impact our strongest findings (for age and sex), as these were objectively assessed. Additionally, as in other studies including genealogical information from registries (without genetic validation), the pedigrees are unlikely to be completely accurate due, for example, to false paternity. A further consideration is that some environmental exposures assessed here are to a limited extent influenced by genetic background [50, 51]; hence, it is possible that what might on the surface appear to be a genotype–environment interaction reflects, at least in part, epistasis.

In conclusion, our results suggest that cardiometabolic traits are heavily influenced by the interactions between the genotype and environmental exposures. Our data indicate that future studies focused on identifying specific genetic variants underlying genotype–environment interactions should focus on the exposures of age, sex and alcohol intake on body composition. Numerous other exposures and outcomes defined here are also plausible candidates for genotype–environment interaction.

## Electronic supplementary material

Below is the link to the electronic supplementary material.

ESM (PDF 4575 kb)

## Abbreviations

E%: Per cent of total energy intake
DBP: Diastolic blood pressure
HDL-C: HDL-cholesterol
LDL-C: LDL-cholesterol
LRT: Likelihood Ratio Test
MUFA: Monounsaturated fatty acids
PUFA: Polyunsaturated fatty acids
SBP: Systolic blood pressure
SOLAR: Sequential Oligogenic Linkage Analysis Routines
VIKING: Västerbotten Imputation Databank of Near-Complete Genomes
