# Supplementary material for: The heritable basis of gene–environment interactions in cardiometabolic traits
Source: Diabetologia. 2016 Dec 21;60(3):442–52. doi: 10.1007/s00125-016-4184-0 (PMC6518092; doi:10.1007/s00125-016-4184-0)
Supplement: Supplementary file 1 — (PDF 4575 kb) [file 125_2016_4184_MOESM1_ESM.pdf]

**ELECTRONIC SUPPLEMENTARY MATERIAL**

**TABLE OF CONTENTS**


| | |
|---|---|
| **ESM Table 1.** Number of relationships between pairs of phenotyped individuals | Page 2 |
| **ESM Table 2.** Principal component analysis of macronutrient intake | Page 3 |
| **ESM Table 3.** Variance components parameters estimates for genotype-age interactions | Page 4 |
| **ESM Table 4.** Variance components parameters estimates (±s.e.) for genotype-sex interactions | Page 5 |
| **ESM Table 5.** Variance components parameters estimates (±s.e.) for genotype-physical activity interactions | Page 6 |
| **ESM Table 6.** Variance components parameters estimates (±s.e.) for genotype-smoking status interactions | Page 7 |
| **ESM Table 7.** Variance components parameters estimates for genotype-alcohol intake interactions | Page 8 |
| **ESM Table 8.** Variance components parameters estimates for genotype-diet pattern interactions | Page 9 |
| **ESM Table 9.** Variance components parameters estimates for genotype-carbohydrate intake interactions | Page 10 |
| **ESM Table 10.** Variance components parameters estimates for genotype-protein intake interactions | Page 11 |
| **ESM Table 11.** Variance components parameters estimates for genotype-fiber intake interactions | Page 12 |
| **ESM Table 12.** Variance components parameters estimates for genotype-total fat intake interactions | Page 13 |
| **ESM Table 13.** Variance components parameters estimates for genotype-saturated fat intake interactions | Page 14 |
| **ESM Table 14.** Variance components parameters estimates for genotype-essential fatty acids intake interactions | Page 15 |
| **ESM Table 15.** Variance components parameters estimates for genotype-polyunsaturated fat intake interactions | Page 16 |
| **ESM Table 16.** Variance components parameters estimates for genotype-monounsaturated fat intake interactions | Page 17 |
| **ESM Table 17.** Sensitivity analyses for glycaemic and lipid traits excluding non-fasted individuals | Pages 18-32 |
| **ESM Figure 1.** *Class 1* genotype-physical activity interactions | Page 33 |
| **ESM Figure 2.** Genotype-diet components interactions | Pages 34-35 |
| **ESM Figure 3.** Genotype-fat interactions | Pages 36-37 |

## ELECTRONIC SUPPLEMENTARY TABLES

**ESM Table 1.** Number of relationships between pairs of phenotyped individuals

| Kinship coefficient (Φ; range) | Relationship | Number of pairs |
|---|---|---|
| 0.25 (0.20≤Φ<0.30) | First-degree relatives (PO, FS) | 8,908 |
| 0.125 (0.10≤Φ<0.20) | Second-degree relatives (HS, AV, GG, D1C) | 5,794 |
| 0.0625 (0.05≤Φ<0.10) | Third-degree relatives (1C, GAV, GGG) | 29,706 |
| 0.03125 (0.02≤Φ<0.05) | Fourth-degree relatives (1.5C, D2C) | 45,939 |
| 0.01563 (0.01≤Φ<0.02) | Fifth-degree relative (2C) | 131,122 |
| 0.00781 (0.005≤Φ<0.01) | Sixth-degree relative (2.5C) | 335,519 |
| 0≤Φ<0.005 | Others | 37.640,640 |

PO, parent–offspring; FS, full sibling; HS, half sibling; AV, avuncular; GG, grandparent–grandchild; D1C, double first cousins; 1C, first cousins; GAV, grand-avuncular; GGG, great grandparent-great grandchild; 1.5C, first cousin once removed; D2C, double second cousin; 2C, second cousin; 2.5C, second cousin once removed.

**ESM Table 2.** Principal component analysis of macronutrient intake

| Macronutrients intake | Factor loading |
|---|---|
| Carbohydrate | -0.92 |
| Protein | -0.10 |
| Total fat | 0.97 |
| Saturated fat | 0.80 |
| MUFA | 0.93 |
| PUFA | 0.57 |
| Essential fatty acids | 0.48 |
| Fibre | -0.66 |
| | |
| Eigenvalue | 4.3 |
| Percent of total variance | 53.8% |

MUFA, monounsaturated fatty acids; PUFA, polyunsaturated fatty acids. Essential fatty acids: omega-3 and omega-6 fatty acids.

**ESM Table 3.** Variance components parameters estimates for genotype-age interactions on 11 cardiometabolic traits

| Phenotypes | $N$ | $\alpha_G$ | $\gamma_G$ | λ | $\alpha_e$ | $\gamma_e$ | $\gamma_G$ *p-value* | λ *p-value* |
|---|---|---|---|---|---|---|---|---|
| Weight, kg | 16,296 | -0.7763 | -0.0055 | 0.0242 | -0.6414 | 0.0146 | 0.35 | **$2\times10^{-6}$*** |
| BMI, kg/m$^2$ | 16,281 | -0.8220 | 0.0012 | 0.0145 | -0.6011 | 0.0219 | 0.85 | **$3\times10^{-3}$** |
| Waist circumference, cm | 1,905 | -0.7502 | -0.0198 | 0 | -0.6645 | 0.0221 | 0.59 | 0.50 |
| TC, mmol/l | 14,366 | -0.8409 | 0.0036 | 0.0177 | -0.5873 | 0.0131 | 0.66 | **$3\times10^{-3}$** |
| HDL-C, mmol/l | 3,266 | -0.7780 | -0.0027 | 0.0252 | -0.6254 | 0.0094 | 0.79 | 0.06 |
| LDL-C, mmol/l | 3,169 | -0.5760 | 0.0079 | 0.0393 | -0.8441 | 0.0144 | 0.39 | **$1\times10^{-2}$** |
| TG, mmol/l | 11,681 | -1.0485 | 0.0075 | 0 | -0.4405 | 0.0047 | 0.33 | 0.50 |
| Fasting glucose, mmol/l | 14,507 | -1.3952 | 0.0593 | 0 | -0.3294 | 0.0063 | **$1\times10^{-2}$** | 0.50 |
| 2 h glucose, mmol/l | 13,603 | -1.2873 | -0.0255 | 0.0207 | -0.3827 | 0.0404 | **$3\times10^{-2}$** | **$3\times10^{-2}$** |
| SBP, mmHg | 16,097 | -1.0200 | 0.0518 | 0.0253 | -0.5231 | 0.0334 | **$<1\times10^{-9}$*** | **$1\times10^{-3}$** |
| DBP, mmHg | 16,092 | -1.0881 | 0.0189 | 0.0188 | -0.4225 | 0.0138 | **$9\times10^{-3}$** | **$1\times10^{-2}$** |

Significant estimates are marked in bold. * Withstand the Bonferroni corrected *p-value* of 0.00019.

TC, total cholesterol; TG, triacylglycerol.

$\alpha_G$, $\gamma_G$, $\lambda$, $\alpha_e$ and $\gamma_e$ are parameters estimated in the genotype-environment interaction model for continuous environmental exposures (see Eq. 4 and 5). $\gamma_G$ *p-value*: *p-value* of the variance homogeneity null hypothesis; $\lambda$ *p-value*: *p-value* of the genetic correlation equal to 1 null hypothesis.

**ESM Table 4.** Variance components parameters estimates (±s.e.) for genotype-sex interactions on 11 cardiometabolic traits

| Phenotypes | *N* | $\sigma_G$ Females | $\sigma_G$ Males | $\sigma_E$ Females | $\sigma_E$ Males | $\sigma$ *p*-value | $\rho_G$ | $\rho_G$ *p*-value |
|---|---|---|---|---|---|---|---|---|
| Weight, kg | 16,296 | 0.63±0.03 | 0.60±0.03 | 0.76±0.02 | 0.80±0.02 | 0.37 | 0.86±0.08 | **$5\times10^{-2}$** |
| BMI, $kg/m^2$ | 16,281 | 0.72±0.03 | 0.53±0.03 | 0.81±0.02 | 0.72±0.02 | **$4\times10^{-6}$*** | 0.89±0.08 | 0.08 |
| Waist circumference, cm | 1,905 | 0.83±0.20 | 0.60±0.18 | 0.72±0.23 | 0.68±0.16 | 0.40 | 0.85±0.50 | 0.39 |
| TC, mmol/l | 14,366 | 0.66±0.03 | 0.61±0.03 | 0.74±0.02 | 0.79±0.02 | 0.24 | 0.79±0.08 | **$8\times10^{-3}$** |
| HDL-C, mmol/l | 3,266 | 0.61±0.11 | 0.67±0.08 | 0.86±0.08 | 0.66±0.08 | 0.65 | 0.80±0.26 | 0.22 |
| LDL-C, mmol/l | 3,169 | 0.81±0.08 | 0.48±0.09 | 0.56±0.10 | 0.89±0.05 | **$2\times10^{-2}$** | 1 | - |
| TG, mmol/l | 11,681 | 0.54±0.03 | 0.82±0.04 | 0.61±0.02 | 0.83±0.04 | **$4\times10^{-7}$*** | 0.55±0.07 | **$2\times10^{-10}$*** |
| Fasting glucose, mmol/l | 14,507 | 0.53±0.03 | 0.51±0.04 | 0.81±0.02 | 0.89±0.02 | 0.67 | 0.73±0.13 | **$3\times10^{-2}$** |
| 2 h glucose, mmol/l | 13,603 | 0.54±0.04 | 0.53±0.04 | 0.80±0.03 | 0.89±0.03 | 0.93 | 0.77±0.14 | 0.06 |
| SBP, mmHg | 16,097 | 0.67±0.03 | 0.52±0.03 | 0.77±0.03 | 0.82±0.02 | **$2\times10^{-3}$** | 0.84±0.08 | **$3\times10^{-2}$** |
| DBP, mmHg | 16,092 | 0.61±0.03 | 0.51±0.04 | 0.78±0.02 | 0.87±0.02 | **$4\times10^{-2}$** | 0.92±0.10 | 0.21 |

Significant estimates are marked in bold. * Withstand the Bonferroni corrected *P*-value of 0.00019.

TC, total cholesterol; TG, triacylglycerol.

$\sigma_G$: Genetic standard deviation; $\sigma_E$: Environmental standard deviation; $\sigma$ *p*-value: *p*-value of the variance homogeneity null hypothesis; $\rho_G$: genetic correlation; $\rho_G$ *p*-value: *p*-value of the genetic correlation equal to 1 null hypothesis.

**ESM Table 5.** Variance components parameters estimates (±s.e.) for genotype-physical activity interactions on 11 cardiometabolic traits

| Phenotypes | *N* | $\sigma_G$ Active | $\sigma_G$ Inactive | $\sigma_E$ Active | $\sigma_E$ Inactive | $\sigma$ *p*-value | $\rho_G$ | $\rho_G$ *p*-value |
|---|---|---|---|---|---|---|---|---|
| Weight, kg | 14,350 | 0.60±0.05 | 0.70±0.02 | 0.71±0.04 | 0.75±0.02 | 0.06 | 0.99±0.10 | 0.45 |
| BMI, kg/m$^2$ | 14,341 | 0.61±0.05 | 0.68±0.03 | 0.69±0.04 | 0.77±0.02 | 0.17 | 0.92±0.10 | 0.21 |
| Waist circumference, cm | 1,846 | 0.37±0.20 | 0.79±0.12 | 0.84±0.09 | 0.66±0.14 | 0.08 | 1 | - |
| TC, mmol/l | 12,828 | 0.71±0.05 | 0.62±0.03 | 0.70±0.04 | 0.78±0.02 | 0.14 | 1 | - |
| HDL-C, mmol/l | 2,296 | 0.55±0.21 | 0.68±0.13 | 0.82±0.14 | 0.74±0.11 | 0.63 | 1 | - |
| LDL-C, mmol/l | 2,271 | 0.78±0.20 | 0.57±0.12 | 0.59±0.26 | 0.83±0.09 | 0.44 | 1 | - |
| TG, mmol/l | 10,446 | 0.62±0.06 | 0.63±0.03 | 0.61±0.05 | 0.83±0.02 | 0.91 | 0.77±0.11 | **$3\times10^{-2}$** |
| Fasting glucose, mmol/l | 12,831 | 0.50±0.06 | 0.49±0.03 | 0.86±0.03 | 0.87±0.02 | 0.97 | 1 | - |
| 2 h glucose, mmol/l | 12,133 | 0.38±0.06 | 0.54±0.04 | 0.87±0.03 | 0.86±0.02 | **$4\times10^{-2}$** | 1 | - |
| SBP, mmHg | 14,153 | 0.51±0.06 | 0.63±0.03 | 0.79±0.04 | 0.81±0.02 | 0.07 | 0.86±0.14 | 0.17 |
| DBP, mmHg | 14,148 | 0.43±0.08 | 0.61±0.03 | 0.85±0.04 | 0.82±0.02 | **$2\times10^{-2}$** | 0.95±0.21 | 0.40 |

Significant estimates are marked in bold. None of the results withstand the Bonferroni corrected *p*-value of 0.00019.

TC, total cholesterol; TG, triacylglycerol. Physical activity variable was categorized on two categories (inactive and active).

$\sigma_G$: Genetic standard deviation; $\sigma_E$: Environmental standard deviation; $\sigma$ *p*-value: *p*-value of the variance homogeneity null hypothesis; $\rho_G$: genetic correlation; $\rho_G$ *p*-value: *p*-value of the genetic correlation equal to 1 null hypothesis.

**ESM Table 6.** Variance components parameters estimates (±s.e.) for genotype-smoking status interactions on 11 cardiometabolic traits

| Phenotypes | *N* | $\sigma_G$ Non-smokers | $\sigma_G$ Smokers | $\sigma_E$ Non-smokers | $\sigma_E$ Smokers | $\sigma$ *p*-value | $\rho_G$ | $\rho_G$ *p*-value |
|---|---|---|---|---|---|---|---|---|
| Weight, kg | 10,677 | 0.69±0.03 | 0.69±0.05 | 0.72±0.03 | 0.71±0.05 | 0.90 | 0.79±0.11 | **$4\times10^{-2}$** |
| BMI, $kg/m^2$ | 10,668 | 0.67±0.03 | 0.65±0.05 | 0.74±0.03 | 0.75±0.05 | 0.67 | 0.84±0.12 | 0.09 |
| Waist circumference, cm | 1,143 | 0.01±0.33 | 0.95±0.26 | 0.98±0.03 | 0.38±0.65 | 0.17 | 1 | - |
| TC, mmol/l | 9,423 | 0.63±0.03 | 0.65±0.05 | 0.75±0.02 | 0.79±0.04 | 0.70 | 1 | - |
| HDL-C, mmol/l | 1,974 | 0.59±0.12 | 0.60±0.27 | 0.81±0.08 | 0.78±0.20 | 0.98 | 1 | - |
| LDL-C, mmol/l | 1,914 | 0.69±0.11 | 0.64±0.19 | 0.69±0.10 | 0.81±0.15 | 0.82 | 1 | - |
| TG, mmol/l | 7,683 | 0.57±0.04 | 0.60±0.08 | 0.74±0.03 | 0.93±0.05 | 0.76 | 1 | - |
| Fasting glucose, mmol/l | 9,510 | 0.57±0.04 | 0.48±0.09 | 0.81±0.03 | 0.89±0.05 | 0.25 | 0.68±0.24 | 0.12 |
| 2 h glucose, mmol/l | 8,934 | 0.52±0.04 | 0.54±0.08 | 0.84±0.03 | 0.86±0.05 | 0.78 | 0.97±0.24 | 0.46 |
| SBP, mmHg | 10,522 | 0.64±0.03 | 0.57±0.05 | 0.78±0.03 | 0.80±0.04 | 0.31 | 1 | - |
| DBP, mmHg | 10,519 | 0.56±0.04 | 0.56±0.06 | 0.82±0.02 | 0.84±0.04 | 0.98 | 1 | - |

Significant estimates are marked in bold. None of the results withstand the Bonferroni corrected *p*-value of 0.00019.

TC, total cholesterol; TG, triacylglycerol. Smoking status variable was categorized in two categories (smokers and non-smokers).

$\sigma_G$: Genetic standard deviation; $\sigma_E$: Environmental standard deviation; $\sigma$ *p*-value: *p*-value of the variance homogeneity null hypothesis; $\rho_G$: genetic correlation; $\rho_G$ *p*-value: *p*-value of the genetic correlation equal to 1 null hypothesis.

**ESM Table 7.** Variance components parameters estimates for genotype-alcohol intake interactions on 11 cardiometabolic traits

| Phenotypes | $N$ | $\alpha_G$ | $\gamma_G$ | λ | $\alpha_E$ | $\gamma_E$ | $\gamma_G$ $p$-value | λ $p$-value |
|---|---|---|---|---|---|---|---|---|
| Weight, kg | 12,640 | -0.3658 | 0.0501 | 0.1692 | -1.4583 | -0.4680 | **$1\times10^{-5}$*** | **$7\times10^{-7}$*** |
| BMI, kg/m$^2$ | 12,632 | -0.5156 | 0.0432 | 0.1353 | -1.1108 | -0.3938 | **$3\times10^{-4}$** | **$5\times10^{-3}$** |
| Waist circumference, cm | 1,773 | -2.1234 | -0.7836 | 0 | -0.3446 | 0.0303 | 1 | 0.50 |
| TC, mmol/l | 11,300 | -0.8384 | -0.0981 | 0 | -0.5819 | 0.0285 | 0.10 | 0.50 |
| HDL-C, mmol/l | 2,141 | -0.4385 | 0.0617 | 0.1640 | -1.0869 | -0.0831 | 0.30 | 0.11 |
| LDL-C, mmol/l | 2,120 | -0.7464 | -0.0175 | 0.0073 | -0.6546 | -0.0142 | 0.78 | 0.47 |
| TG, mmol/l | 9,797 | -0.9303 | 0.0151 | 0.0543 | -0.5132 | 0.0423 | 0.69 | 0.17 |
| Fasting glucose, mmol/l | 11,298 | -1.4051 | -0.1374 | 0 | -0.2970 | 0.0224 | 0.23 | 0.50 |
| 2 h glucose, mmol/l | 10,675 | -1.5793 | -0.3111 | 0 | -0.2770 | 0.0568 | **$4\times10^{-3}$** | 0.50 |
| SBP, mmHg | 12,464 | -0.9552 | -0.1070 | 0.0964 | -0.5009 | 0.0255 | 0.12 | 0.07 |
| DBP, mmHg | 12,459 | -1.0945 | -0.0555 | 0.0755 | -0.4143 | 0.0325 | 0.36 | 0.14 |

Significant estimates are marked in bold. * Withstand the Bonferroni corrected $p$-value of 0.00019.

TC, total cholesterol; TG, triacylglycerol.

$\alpha_G$, $\gamma_G$, $\lambda$, $\alpha_e$ and $\gamma_e$ are parameters estimated in the genotype-environment interaction model for continuous environmental exposures (see Eq. 4 and 5). $\gamma_G$ $p$-value: $p$-value of the variance homogeneity null hypothesis; $\lambda$ $p$-value: $p$-value of the genetic correlation equal to 1 null hypothesis.

**ESM Table 8.** Variance components parameters estimates for genotype-diet pattern interactions on 11 cardiometabolic traits

| Phenotypes | $N$ | $\alpha_G$ | $\gamma_G$ | λ | $\alpha_E$ | $\gamma_E$ | $\gamma_G$ $p$-value | λ $p$-value |
|---|---|---|---|---|---|---|---|---|
| Weight, kg | 12,640 | -0.5710 | 0.0496 | 0.0446 | -0.8483 | -0.0155 | 0.33 | **$3\times10^{-2}$** |
| BMI, kg/m$^2$ | 12,632 | -0.6104 | 0.0085 | 0.0700 | -0.7873 | -0.0305 | 0.87 | **$1\times10^{-2}$** |
| Waist circumference, cm | 1,773 | -0.2747 | 0.0437 | 0.2306 | -1.6262 | -0.2328 | 0.30 | 0.05 |
| TC, mmol/l | 11,300 | -0.8216 | -0.0568 | 0 | -0.5870 | 0.0187 | 0.18 | 0.50 |
| HDL-C, mmol/l | 2,141 | -0.6773 | 0.0195 | 0 | -0.7263 | -0.0408 | 0.78 | 0.50 |
| LDL-C, mmol/l | 2,120 | -0.8408 | -0.1349 | 0 | -0.6227 | 0.0730 | 0.17 | 0.50 |
| TG, mmol/l | 9,797 | -1.0116 | 0.0687 | 0 | -0.4752 | 0.0619 | 0.27 | 0.50 |
| Fasting glucose, mmol/l | 11,298 | -1.0490 | -0.0890 | 0.1542 | -0.4487 | 0.0429 | 0.13 | **$6\times10^{-3}$** |
| 2 h glucose, mmol/l | 10,675 | -1.1781 | 0.0806 | 0.0993 | -0.3771 | -0.0232 | 0.18 | **$5\times10^{-2}$** |
| SBP, mmHg | 12,464 | -1.0752 | -0.1182 | 0.0004 | -0.4354 | 0.0225 | **$2\times10^{-2}$** | 0.50 |
| DBP, mmHg | 12,459 | -1.2013 | -0.0669 | 0 | -0.3659 | 0.0326 | 0.38 | 0.50 |

Significant estimates are marked in bold. None of the results withstand the Bonferroni corrected $p$-value of 0.00019.

TC, total cholesterol; TG, triacylglycerol.

$\alpha_G$, $\gamma_G$, $\lambda$, $\alpha_E$ and $\gamma_E$ are parameters estimated in the genotype-environment interaction model for continuous environmental exposures (see Eq. 4 and 5). $\gamma_G$ $p$-value: $p$-value of the variance homogeneity null hypothesis; $\lambda$ $p$-value: $p$-value of the genetic correlation equal to 1 null hypothesis.

**ESM Table 9.** Variance components parameters estimates for genotype-carbohydrate intake interactions on 11 cardiometabolic traits

| Phenotypes | $N$ | $\alpha_G$ | $\gamma_G$ | λ | $\alpha_E$ | $\gamma_E$ | $\gamma_G$ $p$-value | λ $p$-value |
|---|---|---|---|---|---|---|---|---|
| Weight, kg | 12,640 | -0.5892 | -0.0050 | 0.0111 | -0.8187 | -0.0074 | 0.78 | 0.08 |
| BMI, kg/m$^2$ | 12,632 | -0.6357 | 0.0130 | 0.0184 | -0.7614 | -0.0119 | 0.46 | **$2\times10^{-2}$** |
| Waist circumference, cm | 1,773 | -0.2298 | -0.0054 | 0.1029 | -1.6915 | 0.0462 | 0.66 | **$2\times10^{-2}$** |
| TC, mmol/l | 11,300 | -0.8269 | 0.0220 | 0 | -0.5848 | -0.0053 | 0.06 | 0.50 |
| HDL-C, mmol/l | 2,141 | -0.5920 | -0.0030 | 0.0055 | -0.8209 | 0.0088 | 0.88 | 0.45 |
| LDL-C, mmol/l | 2,120 | -0.8437 | 0.0530 | 0 | -0.6435 | -0.0280 | **$4\times10^{-2}$** | 0.50 |
| TG, mmol/l | 9,797 | -1.0175 | -0.0117 | 0 | -0.4746 | -0.0270 | 0.56 | 0.50 |
| Fasting glucose, mmol/l | 11,298 | -1.0650 | 0.0185 | 0.0490 | -0.4352 | -0.0150 | 0.38 | **$9\times10^{-3}$** |
| 2 h glucose, mmol/l | 10,675 | -1.2305 | -0.0257 | 0.0254 | -0.3532 | 0.0052 | 0.39 | 0.09 |
| SBP, mmHg | 12,464 | -1.0757 | 0.0385 | 0 | -0.4364 | -0.0116 | **$1\times10^{-2}$** | 0.50 |
| DBP, mmHg | 12,459 | -1.2057 | 0.0300 | 0 | -0.3694 | -0.0145 | 0.13 | 0.50 |

Significant estimates are marked in bold. None of the results withstand the Bonferroni corrected $p$-value of 0.00019.

TC, total cholesterol; TG, triacylglycerol.

$\alpha_G$, $\gamma_G$, $\lambda$, $\alpha_E$ and $\gamma_E$ are parameters estimated in the genotype-environment interaction model for continuous environmental exposures (see Eq. 4 and 5). $\gamma_G$ $p$-value: $p$-value of the variance homogeneity null hypothesis; $\lambda$ $p$-value: $p$-value of the genetic correlation equal to 1 null hypothesis.

**ESM Table 10.** Variance components parameters estimates for genotype-protein intake interactions on 11 cardiometabolic traits

| Phenotypes | $N$ | $\alpha_G$ | $\gamma_G$ | λ | $\alpha_E$ | $\gamma_E$ | $\gamma_G$ $p$-value | λ $p$-value |
|---|---|---|---|---|---|---|---|---|
| Weight, kg | 12,640 | -0.5469 | 0.0633 | 0.0541 | -0.8847 | -0.0163 | 0.48 | $\mathbf{1\times10^{-2}}$ |
| BMI, kg/m$^2$ | 12,632 | -0.6410 | 0.0790 | 0.0607 | -0.7697 | 0.0369 | 0.07 | $\mathbf{2\times10^{-2}}$ |
| Waist circumference, cm | 1,773 | -0.8454 | 0.0371 | 0.0206 | -0.5888 | 0.0589 | 0.59 | 0.45 |
| TC, mmol/l | 11,300 | -0.7721 | -0.0590 | 0.0242 | -0.6307 | 0.0326 | 0.20 | 0.22 |
| HDL-C, mmol/l | 2,141 | -0.1986 | -0.0029 | 0.2265 | -1.7454 | -0.0090 | 0.95 | 0.06 |
| LDL-C, mmol/l | 2,120 | -0.8343 | 0.0546 | 0 | -0.6071 | -0.0875 | 0.28 | 0.50 |
| TG, mmol/l | 9,797 | -1.0054 | 0.1508 | 0 | -0.4986 | -0.0129 | $\mathbf{1\times10^{-2}}$ | 0.50 |
| Fasting glucose, mmol/l | 11,298 | -1.2205 | 0.1154 | 0.0633 | -0.3687 | -0.0082 | 0.33 | 0.18 |
| 2 h glucose, mmol/l | 10,675 | -1.2601 | -0.1374 | 0.0752 | -0.3600 | 0.0584 | $\mathbf{3\times10^{-2}}$ | 0.11 |
| SBP, mmHg | 12,464 | -1.0134 | 0.0565 | 0.0228 | -0.4570 | 0.0060 | 0.36 | 0.27 |
| DBP, mmHg | 12,459 | -1.1653 | 0.0476 | 0.0153 | -0.3787 | -0.0176 | 0.55 | 0.36 |

Significant estimates are marked in bold. None of the results withstand the Bonferroni corrected $p$-value of 0.00019.

TC, total cholesterol; TG, triacylglycerol.

$\alpha_G$, $\gamma_G$, $\lambda$, $\alpha_E$ and $\gamma_E$ are parameters estimated in the genotype-environment interaction model for continuous environmental exposures (see Eq. 4 and 5). $\gamma_G$ $p$-value: $p$-value of the variance homogeneity null hypothesis; $\lambda$ $p$-value: $p$-value of the genetic correlation equal to 1 null hypothesis.

**ESM Table 11.** Variance components parameters estimates for genotype-fibre intake interactions on 11 cardiometabolic traits

| Phenotypes | $N$ | $\alpha_G$ | $\gamma_G$ | λ | $\alpha_E$ | $\gamma_E$ | $\gamma_G$ $p$-value | λ $p$-value |
|---|---|---|---|---|---|---|---|---|
| Weight, kg | 12,640 | -0.6622 | -0.0884 | 0 | -0.7362 | 0.1194 | 0.50 | 0.50 |
| BMI, kg/m$^2$ | 12,632 | -0.7489 | 0.1351 | 0.0065 | -0.6484 | 0.1310 | 0.40 | 0.47 |
| Waist circumference, cm | 1,773 | -0.4229 | 0.0385 | 0.4968 | -1.1072 | 0.2548 | 0.83 | 0.14 |
| TC, mmol/l | 11,300 | -0.7805 | 0.0545 | 0.0429 | -0.6149 | 0.0420 | 0.71 | 0.34 |
| HDL-C, mmol/l | 2,141 | -0.5287 | 0.0802 | 0.2652 | -0.9063 | 0.0274 | 0.64 | 0.32 |
| LDL-C, mmol/l | 2,120 | -0.7305 | -0.0638 | 0 | -0.6740 | 0.1548 | 0.82 | 0.50 |
| TG, mmol/l | 9,797 | -0.9766 | 0.1316 | 0.0827 | -0.5140 | -0.4016 | 0.26 | 0.25 |
| Fasting glucose, mmol/l | 11,298 | -1.3461 | 0.2015 | 0.0192 | -0.3134 | 0.0984 | 0.41 | 0.45 |
| 2 h glucose, mmol/l | 10,675 | -1.2774 | -0.2798 | 0.1916 | -0.3360 | 0.1006 | 0.37 | 0.23 |
| SBP, mmHg | 12,464 | -1.0820 | 0.4028 | 0 | -0.4338 | -0.0353 | **2×10$^{-3}$** | 0.50 |
| DBP, mmHg | 12,459 | -1.2068 | 0.2737 | 0 | -0.3677 | -0.1398 | 0.08 | 0.50 |

Significant estimates are marked in bold. None of the results withstand the Bonferroni corrected $p$-value of 0.00019.

TC, total cholesterol; TG, triacylglycerol.

$\alpha_G$, $\gamma_G$, $\lambda$, $\alpha_E$ and $\gamma_E$ are parameters estimated in the genotype-environment interaction model for continuous environmental exposures (see Eq. 4 and 5). $\gamma_G$ $p$-value: $p$-value of the variance homogeneity null hypothesis; $\lambda$ $p$-value: $p$-value of the genetic correlation equal to 1 null hypothesis.

**ESM Table 12.** Variance components parameters estimates for genotype-total fat intake interactions on 11 cardiometabolic traits

| Phenotypes | $N$ | $\alpha_G$ | $\gamma_G$ | λ | $\alpha_E$ | $\gamma_E$ | $\gamma_G$ $p$-value | λ $p$-value |
|---|---|---|---|---|---|---|---|---|
| Weight, kg | 12,640 | -0.5691 | 0.0194 | 0.0152 | -0.8546 | -0.0103 | 0.14 | **$2\times10^{-2}$** |
| BMI, kg/m$^2$ | 12,632 | -0.5984 | 0.0088 | 0.0261 | -0.8079 | -0.0183 | 0.52 | **$3\times10^{-3}$** |
| Waist circumference, cm | 1,773 | -0.3612 | 0.0163 | 0.0562 | -1.3263 | -0.0618 | 0.27 | 0.1 |
| TC, mmol/l | 11,300 | -0.8185 | -0.0140 | 0 | -0.5867 | 0.0048 | 0.27 | 0.50 |
| HDL-C, mmol/l | 2,141 | -0.6748 | 0.0012 | 0 | -0.7288 | -0.0157 | 0.95 | 0.50 |
| LDL-C, mmol/l | 2,120 | -0.8026 | -0.0347 | 0 | -0.6344 | 0.0191 | 0.39 | 0.50 |
| TG, mmol/l | 9,797 | -1.0060 | 0.0191 | 0 | -0.4737 | 0.0189 | 0.16 | 0.50 |
| Fasting glucose, mmol/l | 11,298 | -1.0811 | -0.0272 | 0.0453 | -0.4315 | 0.0174 | 0.22 | **$1\times10^{-2}$** |
| 2 h glucose, mmol/l | 10,675 | -1.1759 | 0.0295 | 0.0343 | -0.3788 | -0.0054 | 0.10 | **$4\times10^{-2}$** |
| SBP, mmHg | 12,464 | -1.0707 | -0.0346 | 0 | -0.4340 | 0.0081 | 0.07 | 0.50 |
| DBP, mmHg | 12,459 | -1.1845 | -0.0155 | 0.0018 | -0.3698 | 0.0072 | 0.65 | 0.45 |

Significant estimates are marked in bold. None of the results withstand the Bonferroni corrected $p$-value of 0.00019.

TC, total cholesterol; TG, triacylglycerol.

$\alpha_G$, $\gamma_G$, $\lambda$, $\alpha_E$ and $\gamma_E$ are parameters estimated in the genotype-environment interaction model for continuous environmental exposures (see Eq. 4 and 5). $\gamma_G$ $p$-value: $p$-value of the variance homogeneity null hypothesis; $\lambda$ $p$-value: $p$-value of the genetic correlation equal to 1 null hypothesis.

**ESM Table 13.** Variance components parameters estimates for genotype-saturated fat intake interactions on 11 cardiometabolic traits

| Phenotypes | $N$ | $\alpha_G$ | $\gamma_G$ | λ | $\alpha_E$ | $\gamma_E$ | $\gamma_G$ $p$-value | λ $p$-value |
|---|---|---|---|---|---|---|---|---|
| Weight, kg | 12,640 | -0.5273 | 0.0255 | 0.0411 | -0.9139 | -0.0401 | 0.14 | **$3\times10^{-3}$** |
| BMI, kg/m$^2$ | 12,632 | -0.5786 | 0.0192 | 0.0569 | -0.8502 | -0.0663 | 0.23 | **$4\times10^{-4}$** |
| Waist circumference, cm | 1,773 | -0.6582 | 0.0243 | 0.0260 | -0.7769 | -0.0633 | 0.43 | 0.36 |
| TC, mmol/l | 11,300 | -0.8111 | -0.0063 | 0 | -0.5893 | -0.0030 | 0.82 | 0.50 |
| HDL-C, mmol/l | 2,141 | -0.6645 | -0.0105 | 0 | -0.7376 | -0.0193 | 0.78 | 0.50 |
| LDL-C, mmol/l | 2,120 | -0.7685 | -0.0434 | 0 | -0.6445 | 0.0114 | 0.60 | 0.50 |
| TG, mmol/l | 9,797 | -0.9924 | 0.0063 | 0 | -0.4696 | 0.0080 | 0.77 | 0.50 |
| Fasting glucose, mmol/l | 11,298 | -1.1116 | 0.0670 | 0.0774 | -0.4234 | -0.0354 | **$2\times10^{-2}$** | **$2\times10^{-2}$** |
| 2 h glucose, mmol/l | 10,675 | -1.2858 | 0.0465 | 0.0309 | -0.3306 | -0.0105 | 0.19 | 0.17 |
| SBP, mmHg | 12,464 | -1.0633 | -0.0647 | 0.0026 | -0.4376 | 0.0130 | 0.10 | 0.46 |
| DBP, mmHg | 12,459 | -1.1070 | -0.0231 | 0.0256 | -0.4047 | 0.0080 | 0.63 | 0.19 |

Significant estimates are marked in bold. None of the results withstand the Bonferroni corrected $p$-value of 0.00019.

TC, total cholesterol; TG, triacylglycerol.

$\alpha_G$, $\gamma_G$, $\lambda$, $\alpha_E$ and $\gamma_E$ are parameters estimated in the genotype-environment interaction model for continuous environmental exposures (see Eq. 4 and 5). $\gamma_G$ $p$-value: $p$-value of the variance homogeneity null hypothesis; $\lambda$ $p$-value: $p$-value of the genetic correlation equal to 1 null hypothesis.

**ESM Table 14.** Variance components parameters estimates for genotype-essential fatty acids intake interactions on 11 cardiometabolic traits

| Phenotypes | $N$ | $\alpha_G$ | $\gamma_G$ | λ | $\alpha_E$ | $\gamma_E$ | $\gamma_G$ $p$-value | λ $p$-value |
|---|---|---|---|---|---|---|---|---|
| Weight, kg | 12,640 | -0.6282 | 0.0307 | 0.0229 | -0.7730 | 0.0405 | 0.58 | 0.27 |
| BMI, kg/m$^2$ | 12,632 | -0.7026 | 0.0458 | 0.0359 | -0.6871 | -0.0072 | 0.37 | 0.17 |
| Waist circumference, cm | 1,773 | -0.0672 | 0.0138 | 0.7816 | -3.5709 | -0.8476 | 0.66 | 0.12 |
| TC, mmol/l | 11,300 | -0.8251 | -0.0918 | 0 | -0.5865 | 0.0346 | 0.16 | 0.50 |
| HDL-C, mmol/l | 2,141 | -0.7081 | 0.0487 | 0 | -0.6958 | -0.0243 | 0.72 | 0.50 |
| LDL-C, mmol/l | 2,120 | -0.8031 | -0.0629 | 0 | -0.6103 | 0.0315 | 0.54 | 0.50 |
| TG, mmol/l | 9,797 | -1.0157 | 0.0940 | 0 | -0.4696 | 0.0591 | **$4\times10^{-2}$** | 0.50 |
| Fasting glucose, mmol/l | 11,298 | -1.1138 | -0.1005 | 0.1934 | -0.4090 | 0.0369 | 0.20 | **$3\times10^{-2}$** |
| 2 h glucose, mmol/l | 10,675 | -1.3978 | 0.0720 | 0 | -0.2886 | -0.0226 | 0.37 | 0.50 |
| SBP, mmHg | 12,464 | -1.0518 | 0.0931 | 0.0073 | -0.4422 | -0.0644 | 0.06 | 0.42 |
| DBP, mmHg | 12,459 | -1.1939 | 0.1056 | 0.0048 | -0.3725 | -0.0466 | **$4\times10^{-2}$** | 0.46 |

Significant estimates are marked in bold. None of the results withstand the Bonferroni corrected $p$-value of 0.00019.

TC, total cholesterol; TG, triacylglycerol.

$\alpha_G$, $\gamma_G$, $\lambda$, $\alpha_E$ and $\gamma_E$ are parameters estimated in the genotype-environment interaction model for continuous environmental exposures (see Eq. 4 and 5). $\gamma_G$ $p$-value: $p$-value of the variance homogeneity null hypothesis; $\lambda$ $p$-value: $p$-value of the genetic correlation equal to 1 null hypothesis.

**ESM Table 15.** Variance components parameters estimates for genotype-polyunsaturated fat intake interactions on 11 cardiometabolic traits

| Phenotypes | $N$ | $\alpha_G$ | $\gamma_G$ | $\lambda$ | $\alpha_E$ | $\gamma_E$ | $\gamma_G$ $p$-value | $\lambda$ $p$-value |
|---|---|---|---|---|---|---|---|---|
| Weight, kg | 12,640 | -0.6181 | 0.0338 | 0.0311 | -0.7869 | 0.0529 | 0.37 | 0.18 |
| BMI, kg/m$^2$ | 12,632 | -0.6920 | 0.0372 | 0.0473 | -0.6973 | 0.0163 | 0.32 | 0.11 |
| Waist circumference, cm | 1,773 | -0.6584 | 0.0539 | 0.1357 | -0.7597 | -0.0626 | 0.58 | 0.35 |
| TC, mmol/l | 11,300 | -0.8240 | -0.0796 | 0 | -0.5863 | 0.0372 | 0.19 | 0.50 |
| HDL-C, mmol/l | 2,141 | -0.7137 | 0.0537 | 0 | -0.6918 | -0.0256 | 0.57 | 0.50 |
| LDL-C, mmol/l | 2,120 | -0.8003 | -0.0723 | 0 | -0.6161 | 0.0478 | 0.48 | 0.50 |
| TG, mmol/l | 9,797 | -1.0194 | 0.1066 | 0 | -0.4689 | 0.0495 | **$3\times10^{-2}$** | 0.50 |
| Fasting glucose, mmol/l | 11,298 | -1.0603 | -0.0895 | 0.2241 | -0.4365 | 0.0417 | 0.17 | **$1\times10^{-2}$** |
| 2 h glucose, mmol/l | 10,675 | -1.4079 | 0.0828 | 0 | -0.2863 | -0.0241 | 0.29 | 0.50 |
| SBP, mmHg | 12,464 | -1.0301 | 0.0894 | 0.0214 | -0.4548 | -0.0686 | 0.05 | 0.30 |
| DBP, mmHg | 12,459 | -1.2055 | 0.1083 | 0 | -0.3682 | -0.0482 | **$4\times10^{-2}$** | 0.50 |

Significant estimates are marked in bold. None of the results withstand the Bonferroni corrected $p$-value of 0.00019.

TC, total cholesterol; TG, triacylglycerol.

$\alpha_G$, $\gamma_G$, $\lambda$, $\alpha_E$ and $\gamma_E$ are parameters estimated in the genotype-environment interaction model for continuous environmental exposures (see Eq. 4 and 5). $\gamma_G$ $p$-value: $p$-value of the variance homogeneity null hypothesis; $\lambda$ $p$-value: $p$-value of the genetic correlation equal to 1 null hypothesis.

**ESM Table 16.** Variance components parameters estimates for genotype-monounsaturated fat intake interactions on 11 cardiometabolic traits

| Phenotypes | $N$ | $\alpha_G$ | $\gamma_G$ | λ | $\alpha_E$ | $\gamma_E$ | $\gamma_G$ $p$-value | λ $p$-value |
|---|---|---|---|---|---|---|---|---|
| Weight, kg | 12,640 | -0.5788 | 0.0322 | 0.0366 | -0.8356 | 0.0245 | 0.33 | **$4\times10^{-2}$** |
| BMI, kg/m$^2$ | 12,632 | -0.6489 | 0.0014 | 0.0475 | -0.7412 | 0.0120 | 0.96 | **$3\times10^{-2}$** |
| Waist circumference, cm | 1,773 | -0.2592 | 0.0650 | 0.2758 | -1.7000 | -0.2205 | 0.09 | **$3\times10^{-2}$** |
| TC, mmol/l | 11,300 | -0.8221 | -0.0464 | 0 | -0.5857 | 0.0175 | 0.15 | 0.50 |
| HDL-C, mmol/l | 2,141 | -0.7020 | 0.0300 | 0 | -0.7037 | -0.0387 | 0.63 | 0.50 |
| LDL-C, mmol/l | 2,120 | -0.5298 | -0.0649 | 0.1001 | -0.9257 | 0.0515 | 0.79 | 0.25 |
| TG, mmol/l | 9,797 | -1.0165 | 0.0956 | 0 | -0.4742 | 0.0319 | 0.32 | 0.50 |
| Fasting glucose, mmol/l | 11,298 | -1.1380 | -0.0848 | 0.0961 | -0.4022 | 0.0343 | 0.11 | **$4\times10^{-2}$** |
| 2 h glucose, mmol/l | 10,675 | -1.0631 | 0.0575 | 0.1415 | -0.4321 | -0.0292 | 0.21 | **$6\times10^{-3}$** |
| SBP, mmHg | 12,464 | -0.9912 | -0.0686 | 0.0340 | -0.4727 | -0.0064 | 0.08 | 0.21 |
| DBP, mmHg | 12,459 | -1.1823 | -0.0393 | 0.0066 | -0.3700 | 0.0125 | 0.47 | 0.44 |

Significant estimates are marked in bold. None of the results withstand the Bonferroni corrected $p$-value of 0.00019.

TC, total cholesterol; TG, triacylglycerol.

$\alpha_G$, $\gamma_G$, $\lambda$, $\alpha_E$ and $\gamma_E$ are parameters estimated in the genotype-environment interaction model for continuous environmental exposures (see Eq. 4 and 5). $\gamma_G$ $p$-value: $p$-value of the variance homogeneity null hypothesis; $\lambda$ $p$-value: $p$-value of the genetic correlation equal to 1 null hypothesis.

**ESM Table 17.** Sensitivity analyses for glycaemic and lipid traits excluding non-fasted individuals

a) Heritability estimates ($h^2$) of glycaemic and lipid traits

| Phenotypes | *N* | $h^2$ | S.E. | *p*-value |
|---|---|---|---|---|
| TC, mmol/l | 11,916 | 0.41 | 0.03 | $2\times10^{-64}$ |
| HDL-C, mmol/l | 2,278 | 0.42 | 0.12 | $4\times10^{-4}$ |
| LDL-C, mmol/l | 2,256 | 0.44 | 0.12 | $8\times10^{-5}$ |
| TG, mmol/l | 10,392 | 0.36 | 0.03 | $8\times10^{-42}$ |
| Fasting glucose, mmol/l | 12,043 | 0.27 | 0.03 | $2\times10^{-36}$ |
| 2 h glucose, mmol/l | 11,360 | 0.27 | 0.03 | $2\times10^{-26}$ |

TC, total cholesterol; TG, triacylglycerol.

b) Variance components parameters estimates for genotype-age interactions on glycaemic and lipid traits

| Phenotypes | $N$ | $\alpha_G$ | $\gamma_G$ | λ | $\alpha_E$ | $\gamma_E$ | $\gamma_G$ $p$-value | λ $p$-value |
|---|---|---|---|---|---|---|---|---|
| TC, mmol/l | 11,916 | -0.8155 | 0.0031 | 0.0118 | -0.5961 | 0.0126 | 0.73 | 0.08 |
| HDL-C, mmol/l | 2,278 | -0.9072 | -0.0292 | 0.0005 | -0.543 | 0.0043 | 0.3 | 0.49 |
| LDL-C, mmol/l | 2,256 | -0.8234 | 0.0065 | 0 | -0.589 | 0.0006 | 0.76 | 0.5 |
| TG, mmol/l | 10,392 | -1.034 | 0.006 | 0 | -0.4513 | 0.0034 | 0.43 | 0.5 |
| Fasting glucose, mmol/l | 12,043 | -1.2952 | 0.0232 | 0 | -0.3501 | 0.0223 | 0.35 | 0.5 |
| 2 h glucose, mmol/l | 11,360 | -1.1764 | 0.0297 | 0.0217 | -0.3945 | 0.0224 | 0.08 | 0.07 |

TC, total cholesterol; TG, triacylglycerol.

$\alpha_G$, $\gamma_G$, $\lambda$, $\alpha_E$ and $\gamma_E$ are parameters estimated in the genotype-environment interaction model for continuous environmental exposures (see Eq. 4 and 5). $\gamma_G$ $p$-value: $p$-value of the variance homogeneity null hypothesis; $\lambda$ $p$-value: $p$-value of the genetic correlation equal to 1 null hypothesis.

c) Variance components parameters estimates (±s.e.) for genotype-sex interactions on glycaemic and lipid traits

| Phenotypes | *N* | $\sigma_G$ Females | $\sigma_G$ Males | $\sigma_E$ Females | $\sigma_E$ Males | $\sigma$ *p*-value | $\rho_G$ | $\rho_G$ *p*-value |
|---|---|---|---|---|---|---|---|---|
| TC, mmol/l | 11,916 | 0.66±0.04 | 0.67±0.04 | 0.74±0.03 | 0.74±0.03 | 0.78 | 0.82±0.09 | **$3\times10^{-2}$** |
| HDL-C, mmol/l | 2,278 | 0.77±0.17 | 0.77±0.12 | 0.72±0.18 | 0.53±0.16 | 0.99 | 0.36±0.32 | **$5\times10^{-2}$** |
| LDL-C, mmol/l | 2,256 | 0.68±0.13 | 0.64±0.15 | 0.71±0.13 | 0.79±0.12 | 0.85 | 1 | - |
| TG, mmol/l | 10,392 | 0.57±0.03 | 0.85±0.05 | 0.57±0.03 | 0.82±0.04 | **$2\times10^{-6}$*** | 0.48±0.08 | **5E$^{-11}$*** |
| Fasting glucose, mmol/l | 12,043 | 0.57±0.04 | 0.53±0.05 | 0.79±0.03 | 0.87±0.03 | 0.42 | 0.78±0.14 | 0.07 |
| 2 h glucose, mmol/l | 11,360 | 0.55±0.04 | 0.55±0.05 | 0.78±0.03 | 0.89±0.03 | 0.94 | 0.77±0.15 | 0.08 |

Significant estimates are marked in bold. * Withstand the Bonferroni corrected *p*-value of 0.00019.

TC, total cholesterol; TG, triacylglycerol.

$\sigma_G$: Genetic standard deviation; $\sigma_E$: Environmental standard deviation; $\sigma$ *p*-value: *p*-value of the variance homogeneity null hypothesis; $\rho_G$: genetic correlation; $\rho_G$ *p*-value: *p*-value of the genetic correlation equal to 1 null hypothesis.

d) Variance components parameters estimates (±s.e.) for genotype-physical activity interactions on glycaemic and lipid traits

| Phenotypes | *N* | $\sigma_G$ Active | $\sigma_G$ Inactive | $\sigma_E$ Active | $\sigma_E$ Inactive | $\sigma$ *p*-value | $\rho_G$ | $\rho_G$ *p*-value |
|---|---|---|---|---|---|---|---|---|
| TC, mmol/l | 11,434 | 0.69±0.05 | 0.64±0.03 | 0.71±0.05 | 0.77±0.02 | 0.37 | 1 | - |
| HDL-C, mmol/l | 2,166 | 0.58±0.23 | 0.62±0.14 | 0.80±0.16 | 0.78±0.11 | 0.9 | 1 | - |
| LDL-C, mmol/l | 2,144 | 0.72±0.22 | 0.58±0.13 | 0.65±0.24 | 0.83±0.09 | 0.61 | 1 | - |
| TG, mmol/l | 9,967 | 0.61±0.06 | 0.63±0.03 | 0.61±0.05 | 0.83±0.03 | 0.73 | 0.77±0.11 | **$3\times10^{-2}$** |
| Fasting glucose, mmol/l | 11,434 | 0.56±0.08 | 0.52±0.03 | 0.82±0.05 | 0.86±0.02 | 0.6 | 0.93±0.18 | 0.35 |
| 2 h glucose, mmol/l | 10,810 | 0.37±0.07 | 0.57±0.04 | 0.87±0.03 | 0.85±0.02 | **$1\times10^{-2}$** | 1 | - |

Significant estimates are marked in bold. None of the results withstand the Bonferroni corrected *p*-value of 0.00019.

TC, total cholesterol; TG, triacylglycerol. Physical activity variable was categorized on two categories (inactive and active).

$\sigma_G$: Genetic standard deviation; $\sigma_E$: Environmental standard deviation; $\sigma$ *p*-value: *p*-value of the variance homogeneity null hypothesis; $\rho_G$: genetic correlation; $\rho_G$ *p*-value: *p*-value of the genetic correlation equal to 1 null hypothesis.

e) Variance components parameters estimates (±s.e.) for genotype-smoking status interactions on glycaemic and lipid traits

| Phenotypes | *N* | $\sigma_G$ Non-smokers | $\sigma_G$ Smokers | $\sigma_E$ Non-smokers | $\sigma_E$ Smokers | $\sigma$ *p*-value | $\rho_G$ | $\rho_G$ *p*-value |
|---|---|---|---|---|---|---|---|---|
| TC, mmol/l | 7,941 | 0.65±0.04 | 0.66±0.06 | 0.73±0.03 | 0.79±0.05 | 0.98 | 1 | - |
| HDL-C, mmol/l | 1,456 | 0.65±0.18 | 0.98±0.03 | 0.76±0.15 | 0 | 0.21 | -0.13±0.56 | 0.07 |
| LDL-C, mmol/l | 1,444 | 0.69±0.17 | 0.53±0.27 | 0.68±0.17 | 0.90±0.16 | 0.59 | 1 | - |
| TG, mmol/l | 6,955 | 0.60±0.04 | 0.60±0.08 | 0.71±0.03 | 0.94±0.05 | 0.97 | 1 | - |
| Fasting glucose, mmol/l | 8,018 | 0.61±0.04 | 0.43±0.10 | 0.79±0.03 | 0.90±0.05 | 0.08 | 0.97±0.33 | 0.46 |
| 2 h glucose, mmol/l | 7,559 | 0.52±0.05 | 0.53±0.08 | 0.83±0.03 | 0.88±0.05 | 0.95 | 1 | - |

TC, total cholesterol; TG, triacylglycerol. Smoking status variable was categorized in two categories (smokers and non-smokers).

$\sigma_G$: Genetic standard deviation; $\sigma_E$: Environmental standard deviation; $\sigma$ *p*-value: *p*-value of the variance homogeneity null hypothesis; $\rho_G$: genetic correlation; $\rho_G$ *p*-value: *p*-value of the genetic correlation equal to 1 null hypothesis.

f) Variance components parameters estimates for genotype-alcohol intake interactions on glycaemic and lipid traits

| Phenotypes | $N$ | $\alpha_G$ | $\gamma_G$ | λ | $\alpha_E$ | $\gamma_E$ | $\gamma_G$ $p$-value | λ $p$-value |
|---|---|---|---|---|---|---|---|---|
| TC, mmol/l | 10,650 | -0.8058 | -0.0738 | 0.0275 | -0.6031 | 0.0207 | 0.5 | 0.26 |
| HDL-C, mmol/l | 2,038 | -0.5625 | 0.0686 | 0.143 | -0.8898 | -0.0779 | 0.29 | 0.19 |
| LDL-C, mmol/l | 2,018 | -0.8182 | -0.0147 | 0 | -0.5945 | -0.0165 | 0.83 | 0.5 |
| TG, mmol/l | 9,432 | -0.8951 | 0.0289 | 0.0727 | -0.5379 | 0.0396 | 0.44 | 0.1 |
| Fasting glucose, mmol/l | 10,649 | -1.3892 | -0.1557 | 0 | -0.3059 | 0.0257 | 0.22 | 0.5 |
| 2 h glucose, mmol/l | 10,072 | -1.5676 | -0.313 | 0 | -0.2822 | 0.0582 | **$4{\times}10^{-3}$** | 0.5 |

Significant estimates are marked in bold. None of the results withstand the Bonferroni corrected $p$-value of 0.00019.

TC, total cholesterol; TG, triacylglycerol.

$\alpha_G$, $\gamma_G$, $\lambda$, $\alpha_E$ and $\gamma_E$ are parameters estimated in the genotype-environment interaction model for continuous environmental exposures (see Eq. 4 and 5). $\gamma_G$ $p$-value: $p$-value of the variance homogeneity null hypothesis; $\lambda$ $p$-value: $p$-value of the genetic correlation equal to 1 null hypothesis.

g) Variance components parameters estimates for genotype-diet pattern interactions on glycaemic and lipid traits

| Phenotypes | $N$ | $\alpha_G$ | $\gamma_G$ | λ | $\alpha_E$ | $\gamma_E$ | $\gamma_G$ $p$-value | λ $p$-value |
|---|---|---|---|---|---|---|---|---|
| TC, mmol/l | 10,650 | -0.834 | -0.032 | 0 | -0.574 | -0.0033 | 0.45 | 0.5 |
| HDL-C, mmol/l | 2,038 | -0.7844 | 0.0204 | 0 | -0.6247 | -0.0348 | 0.81 | 0.5 |
| LDL-C, mmol/l | 2,018 | -0.9097 | -0.1352 | 0 | -0.5644 | 0.0554 | 0.29 | 0.5 |
| TG, mmol/l | 9,432 | -1.0102 | 0.0822 | 0 | -0.4796 | 0.063 | 0.17 | 0.5 |
| Fasting glucose, mmol/l | 10,649 | -1.0001 | -0.0811 | 0.1635 | -0.4752 | 0.041 | 0.17 | **$4\times10^{-3}$** |
| 2 h glucose, mmol/l | 10,072 | -1.1269 | 0.0792 | 0.1123 | -0.4013 | -0.0254 | 0.17 | **$4\times10^{-2}$** |

Significant estimates are marked in bold. None of the results withstand the Bonferroni corrected $p$-value of 0.00019.

TC, total cholesterol; TG, triacylglycerol.

$\alpha_G$, $\gamma_G$, $\lambda$, $\alpha_E$ and $\gamma_E$ are parameters estimated in the genotype-environment interaction model for continuous environmental exposures (see Eq. 4 and 5). $\gamma_G$ $p$-value: $p$-value of the variance homogeneity null hypothesis; $\lambda$ $p$-value: $p$-value of the genetic correlation equal to 1 null hypothesis.

h) Variance components parameters estimates for genotype-carbohydrate intake interactions on glycaemic and lipid traits

| Phenotypes | $N$ | $\alpha_G$ | $\gamma_G$ | λ | $\alpha_E$ | $\gamma_E$ | $\gamma_G$ $p$-value | λ $p$-value |
|---|---|---|---|---|---|---|---|---|
| TC, mmol/l | 10,650 | -0.8374 | 0.0156 | 0 | -0.5734 | 0.0003 | 0.18 | 0.5 |
| HDL-C, mmol/l | 2,038 | -0.7388 | -0.0043 | 0 | -0.663 | 0.0075 | 0.86 | 0.5 |
| LDL-C, mmol/l | 2,018 | -0.9024 | 0.0518 | 0 | -0.5877 | -0.0222 | 0.09 | 0.5 |
| TG, mmol/l | 9,432 | -1.0112 | -0.021 | 0 | -0.4784 | -0.0236 | 0.29 | 0.5 |
| Fasting glucose, mmol/l | 10,649 | -1.0409 | 0.0131 | 0.0469 | -0.4448 | -0.0118 | 0.59 | **$1\times10^{-2}$** |
| 2 h glucose, mmol/l | 10,072 | -1.2073 | -0.0225 | 0.0251 | -0.3617 | 0.0043 | 0.41 | 0.1 |

Significant estimates are marked in bold. None of the results withstand the Bonferroni corrected $p$-value of 0.00019.

TC, total cholesterol; TG, triacylglycerol.

$\alpha_G$, $\gamma_G$, $\lambda$, $\alpha_E$ and $\gamma_E$ are parameters estimated in the genotype-environment interaction model for continuous environmental exposures (see Eq. 4 and 5). $\gamma_G$ $p$-value: $p$-value of the variance homogeneity null hypothesis; $\lambda$ $p$-value: $p$-value of the genetic correlation equal to 1 null hypothesis.

i) Variance components parameters estimates for genotype-protein intake interactions on glycaemic and lipid traits

| Phenotypes | $N$ | $\alpha_G$ | $\gamma_G$ | λ | $\alpha_E$ | $\gamma_E$ | $\gamma_G$ $p$-value | λ $p$-value |
|---|---|---|---|---|---|---|---|---|
| TC, mmol/l | 10,650 | -0.7785 | -0.053 | 0.0279 | -0.6249 | 0.0299 | 0.25 | 0.19 |
| HDL-C, mmol/l | 2,038 | -0.0779 | -0.004 | 0.3458 | -2.6808 | -0.0478 | 0.9 | **$1\times10^{-2}$** |
| LDL-C, mmol/l | 2,018 | -0.3889 | 0.0271 | 0.2744 | -1.1926 | -0.1129 | 0.54 | 0.18 |
| TG, mmol/l | 9,432 | -1.0005 | 0.1421 | 0 | -0.497 | -0.007 | **$2\times10^{-2}$** | 0.5 |
| Fasting glucose, mmol/l | 10,649 | -1.2692 | 0.12 | 0.0292 | -0.349 | -0.0062 | 0.18 | 0.29 |
| 2 h glucose, mmol/l | 10,072 | -1.2752 | -0.1338 | 0.0628 | -0.3528 | 0.0572 | 0.05 | 0.15 |

Significant estimates are marked in bold. None of the results withstand the Bonferroni corrected $p$-value of 0.00019.

TC, total cholesterol; TG, triacylglycerol.

$\alpha_G$, $\gamma_G$, $\lambda$, $\alpha_E$ and $\gamma_E$ are parameters estimated in the genotype-environment interaction model for continuous environmental exposures (see Eq. 4 and 5). $\gamma_G$ $p$-value: $p$-value of the variance homogeneity null hypothesis; $\lambda$ $p$-value: $p$-value of the genetic correlation equal to 1 null hypothesis.

j) Variance components parameters estimates for genotype-fibre intake interactions on glycaemic and lipid traits

| Phenotypes | $N$ | $\alpha_G$ | $\gamma_G$ | λ | $\alpha_E$ | $\gamma_E$ | $\gamma_G$ $p$-value | λ $p$-value |
|---|---|---|---|---|---|---|---|---|
| TC, mmol/l | 10,650 | -0.7714 | -0.0117 | 0.0816 | -0.6252 | 0.1016 | 0.94 | 0.25 |
| HDL-C, mmol/l | 2,038 | -0.4597 | 0.0755 | 0.6086 | -1.0178 | 0.0388 | 0.67 | 0.2 |
| LDL-C, mmol/l | 2,018 | -0.8051 | -0.0656 | 0 | -0.6095 | 0.1483 | 0.86 | 0.5 |
| TG, mmol/l | 9,432 | -0.9578 | 0.1013 | 0.1037 | -0.5247 | -0.3979 | 0.4 | 0.2 |
| Fasting glucose, mmol/l | 10,649 | -1.2939 | 0.2016 | 0.0585 | -0.3332 | 0.1018 | 0.34 | 0.36 |
| 2 h glucose, mmol/l | 10,072 | -1.2271 | -0.3054 | 0.2491 | -0.3585 | 0.116 | 0.28 | 0.18 |

TC, total cholesterol; TG, triacylglycerol.

$\alpha_G$, $\gamma_G$, $\lambda$, $\alpha_E$ and $\gamma_E$ are parameters estimated in the genotype-environment interaction model for continuous environmental exposures (see Eq. 4 and 5). $\gamma_G$ $p$-value: $p$-value of the variance homogeneity null hypothesis; $\lambda$ $p$-value: $p$-value of the genetic correlation equal to 1 null hypothesis.

k) Variance components parameters estimates for genotype-total fat intake interactions on glycaemic and lipid traits

| Phenotypes | $N$ | $\alpha_G$ | $\gamma_G$ | λ | $\alpha_E$ | $\gamma_E$ | $\gamma_G$ $p$-value | λ $p$-value |
|---|---|---|---|---|---|---|---|---|
| TC, mmol/l | 10,650 | -0.8324 | -0.0079 | 0 | -0.5745 | -0.001 | 0.51 | 0.5 |
| HDL-C, mmol/l | 2,038 | -0.7808 | 0.003 | 0 | -0.628 | -0.014 | 0.91 | 0.5 |
| LDL-C, mmol/l | 2,018 | -0.8695 | -0.0358 | 0 | -0.5791 | 0.0145 | 0.51 | 0.5 |
| TG, mmol/l | 9,432 | -1.0032 | 0.022 | 0 | -0.4785 | 0.0205 | 0.1 | 0.5 |
| Fasting glucose, mmol/l | 10,649 | -1.0195 | -0.0244 | 0.0507 | -0.4636 | 0.0166 | 0.27 | **$5\times10^{-3}$** |
| 2 h glucose, mmol/l | 10,072 | -1.1278 | 0.0285 | 0.0383 | -0.4013 | -0.0062 | 0.1 | **$3\times10^{-2}$** |

Significant estimates are marked in bold. None of the results withstand the Bonferroni corrected $p$-value of 0.00019.

TC, total cholesterol; TG, triacylglycerol.

$\alpha_G$, $\gamma_G$, $\lambda$, $\alpha_E$ and $\gamma_E$ are parameters estimated in the genotype-environment interaction model for continuous environmental exposures (see Eq. 4 and 5). $\gamma_G$ $p$-value: $p$-value of the variance homogeneity null hypothesis; $\lambda$ $p$-value: $p$-value of the genetic correlation equal to 1 null hypothesis.

l) Variance components parameters estimates for genotype-saturated fat intake interactions on glycaemic and lipid traits

| Phenotypes | $N$ | $\alpha_G$ | $\gamma_G$ | λ | $\alpha_E$ | $\gamma_E$ | $\gamma_G$ $p$-value | λ $p$-value |
|---|---|---|---|---|---|---|---|---|
| TC, mmol/l | 10,650 | -0.8265 | 0.0055 | 0 | -0.5792 | -0.0131 | 0.83 | 0.5 |
| HDL-C, mmol/l | 2,038 | -0.7699 | -0.0044 | 0 | -0.6351 | -0.0167 | 0.92 | 0.5 |
| LDL-C, mmol/l | 2,018 | -0.8296 | -0.036 | 0 | -0.5905 | -0.0009 | 0.72 | 0.5 |
| TG, mmol/l | 9,432 | -0.9766 | 0.0061 | 0.0009 | -0.4792 | 0.0087 | 0.77 | 0.49 |
| Fasting glucose, mmol/l | 10,649 | -1.042 | 0.0605 | 0.0889 | -0.4599 | -0.0388 | **$4\times10^{-2}$** | **$9\times10^{-3}$** |
| 2 h glucose, mmol/l | 10,072 | -1.2132 | 0.0448 | 0.0454 | -0.3607 | -0.0148 | 0.15 | 0.1 |

Significant estimates are marked in bold. None of the results withstand the Bonferroni corrected $p$-value of 0.00019.

TC, total cholesterol; TG, triacylglycerol.

$\alpha_G$, $\gamma_G$, $\lambda$, $\alpha_E$ and $\gamma_E$ are parameters estimated in the genotype-environment interaction model for continuous environmental exposures (see Eq. 4 and 5). $\gamma_G$ $p$-value: $p$-value of the variance homogeneity null hypothesis; $\lambda$ $p$-value: $p$-value of the genetic correlation equal to 1 null hypothesis.

m) Variance components parameters estimates for genotype-essential fatty acids intake interactions on glycaemic and lipid traits

| Phenotypes | $N$ | $\alpha_G$ | $\gamma_G$ | λ | $\alpha_E$ | $\gamma_E$ | $\gamma_G$ $p$-value | λ $p$-value |
|---|---|---|---|---|---|---|---|---|
| TC, mmol/l | 10,650 | -0.8404 | -0.0922 | 0 | -0.5756 | 0.0323 | 0.19 | 0.5 |
| HDL-C, mmol/l | 2,038 | -0.8114 | 0.0045 | 0 | -0.5993 | 0.0089 | 0.96 | 0.5 |
| LDL-C, mmol/l | 2,018 | -0.1718 | -0.0154 | 0.9188 | -1.8899 | 0.0025 | 0.67 | 0.24 |
| TG, mmol/l | 9,432 | -1.012 | 0.1054 | 0 | -0.4749 | 0.0619 | **$2\times10^{-2}$** | 0.5 |
| Fasting glucose, mmol/l | 10,649 | -1.101 | -0.0984 | 0.1827 | -0.416 | 0.0423 | 0.18 | **$4\times10^{-2}$** |
| 2 h glucose, mmol/l | 10,072 | -1.3765 | 0.0558 | 0 | -0.2944 | -0.0117 | 0.55 | 0.5 |

Significant estimates are marked in bold. None of the results withstand the Bonferroni corrected $p$-value of 0.00019.

TC, total cholesterol; TG, triacylglycerol.

$\alpha_G$, $\gamma_G$, $\lambda$, $\alpha_E$ and $\gamma_E$ are parameters estimated in the genotype-environment interaction model for continuous environmental exposures (see Eq. 4 and 5). $\gamma_G$ $p$-value: $p$-value of the variance homogeneity null hypothesis; $\lambda$ $p$-value: $p$-value of the genetic correlation equal to 1 null hypothesis.

n) Variance components parameters estimates for genotype-polyunsaturated fat intake interactions on glycaemic and lipid traits

| Phenotypes | $N$ | $\alpha_G$ | $\gamma_G$ | λ | $\alpha_E$ | $\gamma_E$ | $\gamma_G$ $p$-value | λ $p$-value |
|---|---|---|---|---|---|---|---|---|
| TC, mmol/l | 10,650 | -0.838 | -0.0751 | 0 | -0.5753 | 0.0311 | 0.27 | 0.5 |
| HDL-C, mmol/l | 2,038 | -0.8149 | 0.0193 | 0 | -0.5972 | -0.001 | 0.84 | 0.5 |
| LDL-C, mmol/l | 2,018 | -0.438 | -0.0337 | 0.3454 | -1.0587 | 0.0381 | 0.64 | 0.35 |
| TG, mmol/l | 9,432 | -1.0173 | 0.1161 | 0 | -0.4731 | 0.0527 | **$2\times10^{-2}$** | 0.5 |
| Fasting glucose, mmol/l | 10,649 | -1.0258 | -0.0793 | 0.2299 | -0.4552 | 0.0461 | 0.19 | **$1\times10^{-2}$** |
| 2 h glucose, mmol/l | 10,072 | -1.3859 | 0.0737 | 0 | -0.2926 | -0.0154 | 0.38 | 0.5 |

Significant estimates are marked in bold. None of the results withstand the Bonferroni corrected $p$-value of 0.00019.

TC, total cholesterol; TG, triacylglycerol.

$\alpha_G$, $\gamma_G$, $\lambda$, $\alpha_E$ and $\gamma_E$ are parameters estimated in the genotype-environment interaction model for continuous environmental exposures (see Eq. 4 and 5). $\gamma_G$ $p$-value: $p$-value of the variance homogeneity null hypothesis; $\lambda$ $p$-value: $p$-value of the genetic correlation equal to 1 null hypothesis.

o) Variance components parameters estimates for genotype-monounsaturated fat intake interactions on glycaemic and lipid traits

| Phenotypes | $N$ | $\alpha_G$ | $\gamma_G$ | λ | $\alpha_E$ | $\gamma_E$ | $\gamma_G$ $p$-value | λ $p$-value |
|---|---|---|---|---|---|---|---|---|
| TC, mmol/l | 10,650 | -0.8353 | -0.0323 | 0 | -0.5734 | 0.0019 | 0.31 | 0.5 |
| HDL-C, mmol/l | 2,038 | -0.8074 | 0.0377 | 0 | -0.6094 | -0.0351 | 0.63 | 0.5 |
| LDL-C, mmol/l | 2,018 | -0.4531 | -0.067 | 0.1656 | -1.0576 | 0.0587 | 1 | 0.17 |
| TG, mmol/l | 9,432 | -1.0125 | 0.089 | 0 | -0.477 | 0.042 | 0.22 | 0.5 |
| Fasting glucose, mmol/l | 10,649 | -1.0964 | -0.0704 | 0.1 | -0.4196 | 0.0304 | 0.22 | **$3\times10^{-2}$** |
| 2 h glucose, mmol/l | 10,072 | -0.9906 | 0.0577 | 0.1652 | -0.4737 | -0.0323 | 0.19 | **$3\times10^{-3}$** |

Significant estimates are marked in bold. None of the results withstand the Bonferroni corrected $p$-value of 0.00019.

TC, total cholesterol; TG: triacylglycerol.

$\alpha_G$, $\gamma_G$, $\lambda$, $\alpha_E$ and $\gamma_E$ are parameters estimated in the genotype-environment interaction model for continuous environmental exposures (see Eq. 4 and 5). $\gamma_G$ $p$-value: $p$-value of the variance homogeneity null hypothesis; $\lambda$ $p$-value: $p$-value of the genetic correlation equal to 1 null hypothesis.

## ELECTRONIC SUPPLEMENTARY FIGURES

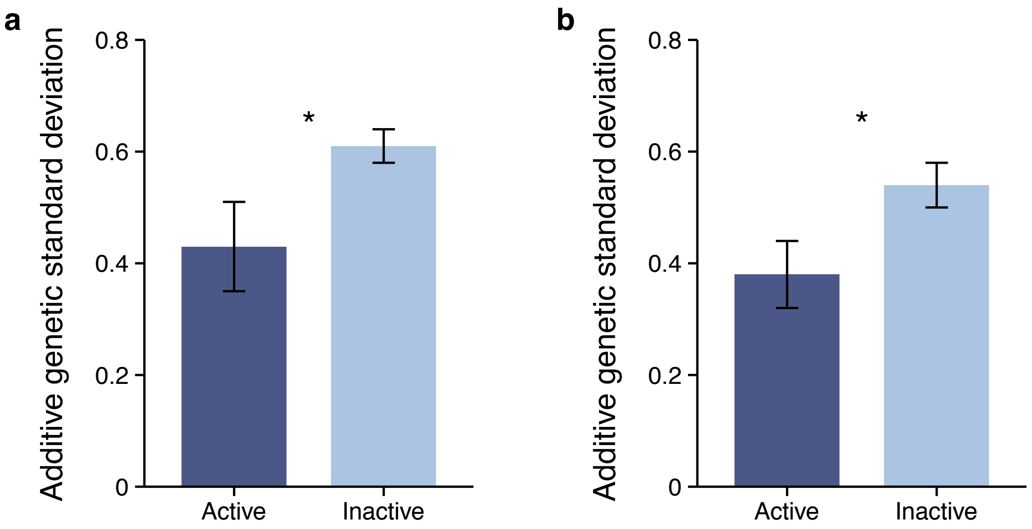


**ESM Figure 1.** ***Class 1*** **genotype-physical activity interactions for a) DBP and b) 2 h glucose**

Only significant traits are represented in the figure; *$p$<0.05, **$p$<0.01, ***$p$<0.001. All interactions were nominally significant ($p$<0.05).

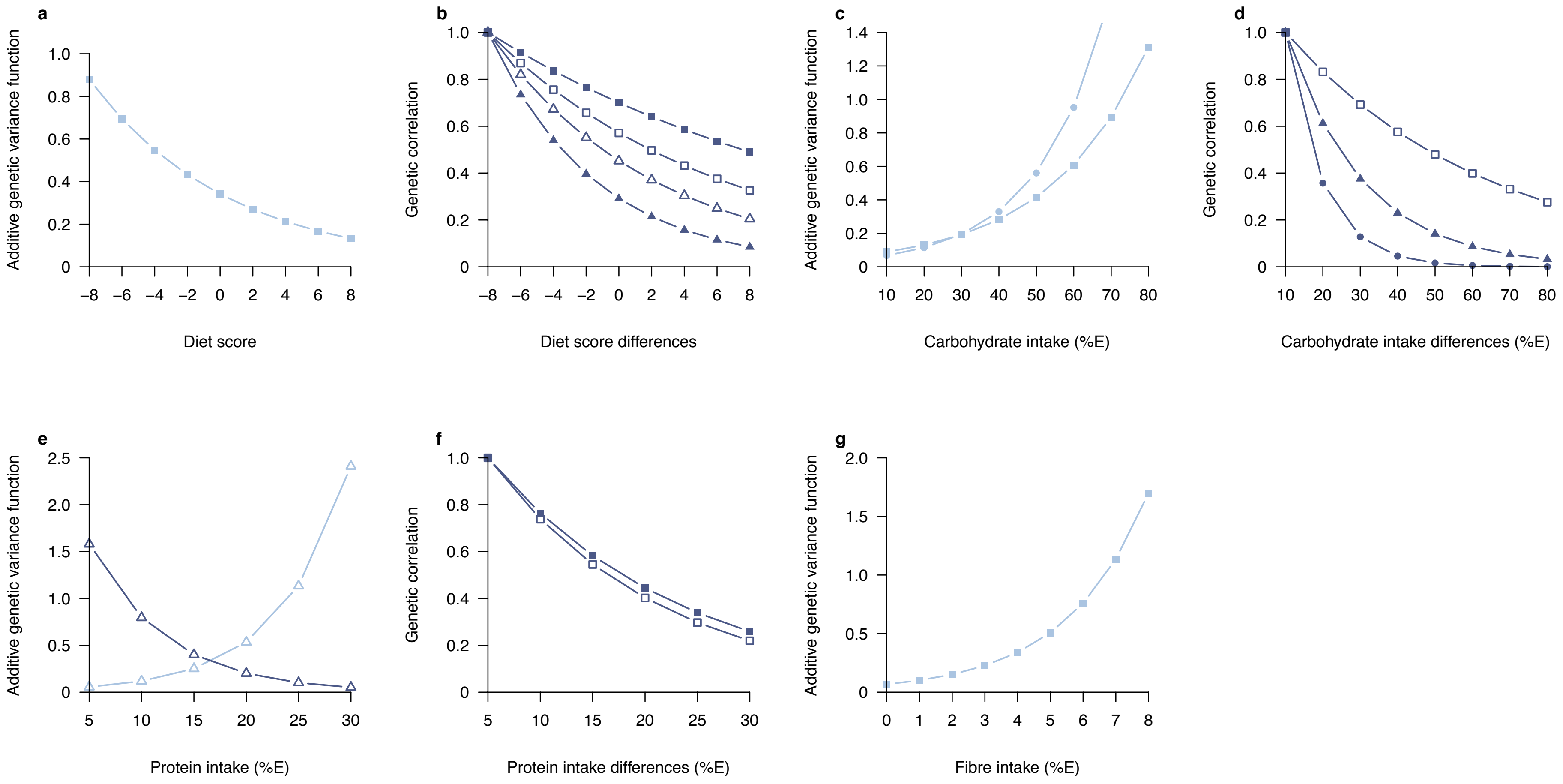
a
b
c
d
e
f
g
Additive genetic variance function
Genetic correlation
Diet score
Diet score differences
Carbohydrate intake (%E)
Carbohydrate intake differences (%E)
Protein intake (%E)
Protein intake differences (%E)
Fibre intake (%E)

**ESM Figure 2. Genotype-diet components interactions**

a) *class 1* genotype-diet score interactions, b) *class 2* genotype-diet score interactions, c) *class 1* genotype-carbohydrate intake interactions, d) *class 2* genotype-carbohydrate intake interactions, e) *class 1* genotype-protein intake interactions, f) *class 2* genotype-protein intake interactions, g) *class 1* genotype-fibre intake interactions.

Dark blue full square, weight; dark blue empty square, BMI; dark blue full circle, waist circumference; dark blue full triangle, fasting glucose; dark blue empty triangle, 2 h glucose; light blue full square, SBP; light blue empty triangle, triacylglycerol; light blue full circle, LDL-C.

Only significant traits are represented in the Figure. All interactions were nominally significant ($p$<0.05).

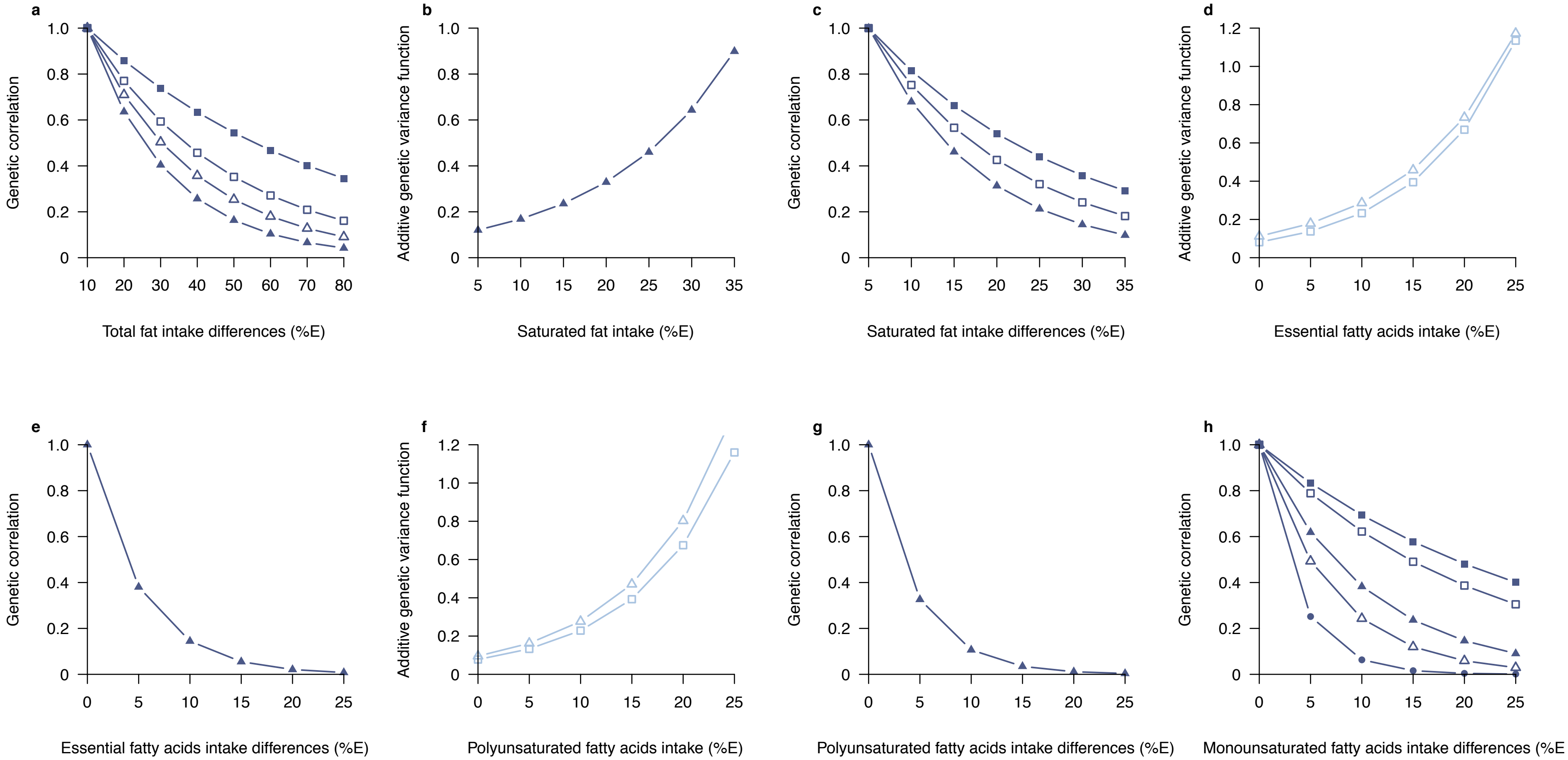

a
Genetic correlation
Total fat intake differences (%E)
b
Additive genetic variance function
Saturated fat intake (%E)
c
Genetic correlation
Saturated fat intake differences (%E)
d
Additive genetic variance function
Essential fatty acids intake (%E)
e
Genetic correlation
Essential fatty acids intake differences (%E)
f
Additive genetic variance function
Polyunsaturated fatty acids intake (%E)
g
Genetic correlation
Polyunsaturated fatty acids intake differences (%E)
h
Genetic correlation
Monounsaturated fatty acids intake differences (%E

**ESM Figure 3. Genotype-fat intake interactions**

a) *class 2* genotype-total fat intake interactions, b) *class 1* genotype-saturated fat intake interactions, c) *class 2* genotype-saturated fat intake interactions, d) *class 1* genotype-essential fatty acid intake interactions, e) *class 2* genotype-essential fatty acid intake interactions, f) *class 1* genotype-polyunsaturated fatty acids intake interactions, g) *class 2* genotype-polyunsaturated fatty acids intake interactions, e) *class 2* genotype-monounsaturated fatty acids intake interactions.

Dark blue full square, weight; dark blue empty square, BMI; dark blue full circle, waist circumference; dark blue full triangle, fasting glucose; dark blue empty triangle, 2 h glucose; light blue empty square, DBP; light blue empty triangle, triacylglycerol.

Only significant traits are represented in the Figure. All interactions were nominally significant ($p$<0.05).
